# Hedgehog Signaling Regulates Neurogenesis in the Larval and Adult Zebrafish Hypothalamus

**DOI:** 10.1523/ENEURO.0226-20.2020

**Published:** 2020-12-18

**Authors:** Ira Male, A. Tuba Ozacar, Rita R. Fagan, Matthew D. Loring, Meng-Chieh Shen, Veronica A. Pace, Christine A. Devine, Grace E. Lawson, Alyssa Lutservitz, Rolf O. Karlstrom

**Affiliations:** Department of Biology, University of Massachusetts, Amherst, 01003 MA

**Keywords:** hedgehog, hypothalamus, neurogenesis, zebrafish

## Abstract

Neurogenesis is now known to play a role in adult hypothalamic function, yet the cell-cell mechanisms regulating this neurogenesis remain poorly understood. Here, we show that Hedgehog (Hh)/Gli signaling positively regulates hypothalamic neurogenesis in both larval and adult zebrafish and is necessary and sufficient for normal hypothalamic proliferation rates. Hh-responsive radial glia represent a relatively highly proliferative precursor population that gives rise to dopaminergic, serotonergic, and GABAergic neurons. *In situ* and transgenic reporter analyses revealed substantial heterogeneity in cell-cell signaling within the hypothalamic niche, with slow cycling Nestin-expressing cells residing among distinct and overlapping populations of Sonic Hh (Shh)-expressing, Hh-responsive, Notch-responsive, and Wnt-responsive radial glia. This work shows for the first time that Hh/Gli signaling is a key component of the complex cell-cell signaling environment that regulates hypothalamic neurogenesis throughout life.

## Visual Abstract

**Figure:**
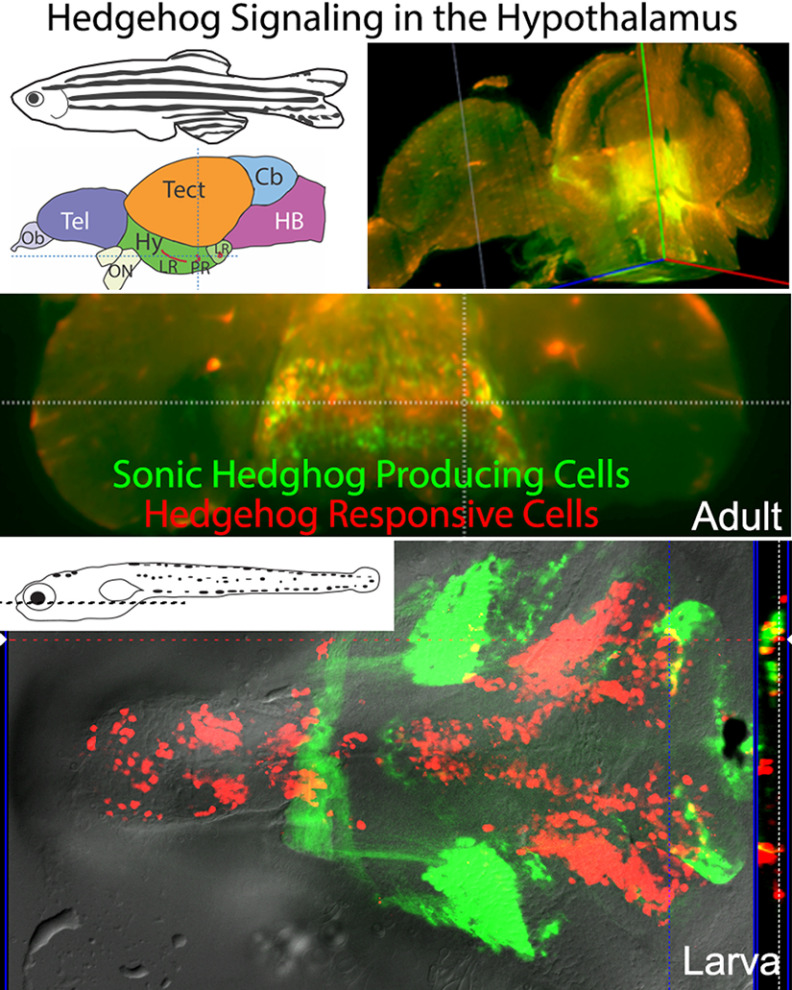

## Significance Statement

The extent, control, and consequences of adult neurogenesis in the hypothalamus are not well understood, despite the critical integrative role this conserved brain region plays in regulating basic metabolic and reproductive functions across vertebrate species. Here, we show that proliferation in the zebrafish hypothalamus continues into adulthood and begin to define the complex signaling environment of the hypothalamic niche that may regulate this adult neurogenesis. Using new conditional gene regulation tools, we show that the evolutionarily conserved Hedgehog (Hh)/Gli signaling pathway positively regulates hypothalamic neurogenesis during postembryonic development and into adulthood. These studies suggest a mechanism for the control of hypothalamic growth and tissue renewal, as well as the plasticity in neuroendocrine cell populations that is now linked to hypothalamic function.

## Introduction

The hypothalamus is among the most ancient and evolutionarily conserved parts of the vertebrate brain (Nieuwenhuys et al., 1998), regulating metabolism, circadian rhythms, autonomic function, and a wide range of behaviors linked to survival (Saper and Lowell, 2014). Dysfunction of the hypothalamus is associated with metabolic and reproductive impairments (Fliers, 2014; Saper and Lowell, 2014), and the hypothalamus is affected in many neurodegenerative disorders (Ishii and Iadecola, 2015; Winner and Winkler, 2015; Vercruysse et al., 2018). Growing evidence indicates that hypothalamic neurogenesis is required for hypothalamic function (Kokoeva et al., 2005; Migaud et al., 2011; Lee et al., 2012; Lee and Blackshaw, 2012; Xie and Dorsky, 2017).

Life-long hypothalamic neurogenesis has now been documented in rodents (Ming and Song, 2011; Yoo and Blackshaw, 2018), sheep (Migaud et al., 2010), zebrafish (Wang et al., 2012; Schmidt et al., 2013), and likely humans (Pellegrino et al., 2018). Similar to more dorsal neurogenic zones, hypothalamic proliferation requires highly coordinated regulation of cell proliferation and differentiation within a discrete population of progenitors. Cell-cell signaling systems that regulate nervous system development such as the Notch, fibroblast growth factor (FGF), Wnt, Hedgehog (Hh), and bone morphogenetic (BMP) signaling pathways play a key role in controlling adult neurogenesis (Kizil et al., 2012; Petrova and Joyner, 2014; Anand and Mondal, 2017; Obernier and Alvarez-Buylla, 2019). Heterogeneity in both neural stem cell populations and in cell-cell signaling systems helps control the range of differentiated cell types that are produced in stem cell niches (März et al., 2010; Chaker et al., 2016; Lim and Alvarez-Buylla, 2016; Ceci et al., 2018). Determining how this heterogeneity contributes to brain growth and adult neurogenesis remains a major challenge in the field.

The highly conserved Hh signaling pathway controls cell proliferation, differentiation, and survival during embryogenesis (Varjosalo and Taipale, 2008; Briscoe and Thérond, 2013) and regulates neural stem cell proliferation in the mammalian hippocampus (Lai et al., 2003; Palma et al., 2005; Álvarez-Buylla and Ihrie, 2014; Petrova and Joyner, 2014; Daynac et al., 2016). Misregulation of Hh signaling is linked to neural tumors including glioblastoma and medulloblastoma (Wechsler-Reya and Scott, 2001; Raleigh and Reiter, 2019) and has been implicated in mediating Parkinson’s disease symptoms and restoring nigrostriatal dopaminergic neurons (Gonzalez-Reyes et al., 2012). While Hh signaling has been shown to be a key component of hypothalamic-pituitary (HP) axis development during embryogenesis (Kondoh et al., 2000; Guner et al., 2008; Blackshaw et al., 2010; Bergeron et al., 2011; Muthu et al., 2016), a role in the postembryonic and adult hypothalamus has not been documented.

The zebrafish brain, which maintains up to 16 proliferative zones throughout life, has proven to be a powerful model for studying adult neurogenesis (Grandel et al., 2006; Schmidt et al., 2013; Anand and Mondal, 2017). Work in the zebrafish has defined a role for notch signaling in the telencephalon (Chapouton et al., 2010, 2011; Rothenaigner et al., 2011) and uncovered a key role for Wnt signaling in hypothalamic neurogenesis, with Wnt signaling being required for the formation of distinct neuronal subtypes and disruptions in Wnt-mediated hypothalamic proliferation leading to reduced growth and behavioral effects (Wang et al., 2012; Duncan et al., 2016; Xie and Dorsky, 2017).

Here, we show that Hh signaling is both necessary and sufficient to regulate hypothalamic neural precursor proliferation throughout the life cycle, with Hh-responsive cells being a previously undescribed population of proliferative radial glia similar to transit amplifying (Type III) neurogenic precursors. Using transgenic reporter lines, we document a diversity of cell signaling responses among radial glia in the ventricular zone. We further demonstrate that Hh-responsive precursors give rise to dopaminergic, GABAergic, and serotonergic neurons. Together, these studies reveal a role for Hh signaling in larval and adult hypothalamic neurogenesis and begin to uncover unrecognized heterogeneity among morphologically similar neural precursors. This work may help explain how a wide range of neuroendocrine and neural populations can be regulated in the adult brain to allow for proper hypothalamic function and HP axis regulation.

## Materials and Methods

### Animals

All animal procedures were performed in accordance with the University of Massachusetts, Amherst Animal Care and Use Committee Regulations. Zebrafish were maintained as groups as in Kimmel et al. (1995). Transgenic lines used are listed in the key resources table (Table 1). Adult sections were taken from females, while larval studies occurred before sex determination.

### Generation of Tet-On Hh-pathway manipulation transgenic lines

Transgene constructs were constructed using Gateway cloning system. The destination and entry clones containing EGFP or mCherry were from the zebrafish Tol2kit systems (Kwan et al., 2007; Villefranc et al., 2007). Entry clones for the Hh-pathway specific elements are described in (Shen et al., 2013) and entry clones with Tet-On system elements were generously provided by the Jensen lab (Campbell et al., 2012). Briefly, 25 pg of purified plasmid DNA was injected to one- to two-cell zebrafish embryos with 25 pg of transposase mRNA. Injected fish were raised to adulthood and out-crossed to wild-type individuals to identify potential founder fish. The transgenic fish containing the *myl7:EGFP* transgene were identified by GFP expression in heart; other lines were PCR genotyped using the primer sets shown in Table 2.

**Table 1:** Key resources

Reagent type (species) or resource	Designation	Source or reference	Identifiers	Additional information
Strain, strain background (*Danio rerio*)	*ptch2:GFP*	Shen et al. (2013)	ZDB-TGCONSTRCT-121112–1	*Tg(GBS-ptch2:EGFP)^umz23Tg^*
Strain, strain background (*Danio rerio*)	*ptch2:NLS-GFP*	Shen et al. (2013)	ZDB-TGCONSTRCT-130123–2	*Tg(GBS-ptch2:NLS-EGFP)^umz24Tg^*
Strain, strain background (*Danio rerio*)	*ptch2:mCherry*	Shen et al. (2013)	ZDB-TGCONSTRCT-130123–4	*Tg(GBS-ptch2:mCherry)^umz26Tg^*
Strain, strain background (*Danio rerio*)	*ptch2:NLS-mCherry*	Shen et al. (2013)	ZDB-TGCONSTRCT-130123–5	*Tg(GBS-ptch2:NLS-mCherry)^umz27Tg^*
Strain, strain background (*Danio rerio*)	*ptch2:kaede*	Huang et al. (2012)	ZDB-TGCONSTRCT-120810–1	*TgBAC(ptch2:Kaede)^a4596Tg^*
Strain, strain background (*Danio rerio*)	*hsp:shha-GFP*	Shen et al. (2013)	ZDB-TGCONSTRCT-130123–8	*Tg(hsp70l:shha-EGFP)^umz30Tg^*
Strain, strain background (*Danio rerio*)	*hsp:gli1-GFP*	Shen et al. (2013)	ZDB-TGCONSTRCT-130123–9	*Tg(hsp70l:gli1-EGFP)^umz31Tg^*
Strain, strain background (*Danio rerio*)	*hsp:gli2aDR-GFP*	Shen et al. (2013)	ZDB-TGCONSTRCT-130123–11	*Tg(hsp70l:gli2aDR-EGFP)^umz33Tg^*
Strain, strain background (*Danio rerio*)	*ptch2:RTTA*	This paper		*Tg(GBS-ptch2:RTTA-HA)^umz39Tg^*
Strain, strain background (*Danio rerio*)	*actb2:RTTA*	This paper		*Tg(actb2:RTTA-HA;myl7:EGFP)^umz40Tg^*
Strain, strain background (*Danio rerio*)	*TETRE:shha-mCherry*	This paper		*Tg(TETRE:shha-mCherry;myl7:EGFP)^umz41Tg^*
Strain, strain background (*Danio rerio*)	*biTETRE:gli1,NLS-mCherry*	This paper		*Tg(biTETRE:gli1,NLS-mCherry)^umz42Tg^*
Strain, strain background (*Danio rerio*)	*biTETRE:gli2aDR,NLS-mCherry*	This paper		*Tg(biTETRE:gli2aDR,NLS-mCherry)^umz43Tg^*
Strain, strain background (*Danio rerio*)	*shha:GFP*	Neumann and Nuesslein-Volhard (2000)	ZDB-TGCONSTRCT-070117–7	*Tg(−2.7shha:GFP)^t10Tg^*
Strain, strain background (*Danio rerio*)	*nes:GFP*	Ganz et al. (2010)	ZDB-TGCONSTRCT-110309–7	*TgBAC(nes:EGFP)^tud100Tg^*
Strain, strain background (*Danio rerio*)	*tp1:GFP*	Parsons et al. (2009)	ZDB-TGCONSTRCT-090625–1	*Tg(EPV.Tp1-Mmu.Hbb:EGFP)^um14Tg^*
Strain, strain background (*Danio rerio*)	*tcf.siam:GFP*	Moro et al. (2012)	ZDB-TGCONSTRCT-110113–1	*Tg(7×TCF-Xla.Sia:GFP)^ia4^*
Strain, strain background (*Danio rerio*)	*dat:GFP*	Xi et al. (2011)	ZDB-TGCONSTRCT-111206–9	*Tg(slc6a3:EGFP)^ot80Tg^*
Strain, strain background (*Danio rerio*)	*vmat2:GFP*	Wen et al. (2008)	ZDB-TGCONSTRCT-170608–1	*Tg(slc18a2:GFP)^zf710Tg^*
Strain, strain background (*Danio rerio*)	*vgluta:DsRed*	Satou et al. (2013)	ZDB-TGCONSTRCT-100505–2	*Tg(slc17a6b:DsRed)^nns9Tg^*
Strain, strain background (*Danio rerio*)	*gfap:NLS-mCh*	Johnson et al. (2016)	ZDB-TGCONSTRCT-160630–1	*Tg(gfap:NLS-mCherry****)***
Strain, strain background (*Danio rerio*)	*gad1b:GFP*	Satou et al. (2013)	ZDB-TGCONSTRCT-131127–6	*TgBAC(gad1b:GFP)^nns25^*
Antibody	Anti-serotonin (rabbit polyclonal)	ImmunostarInc	20080	1:1000
Antibody	Anti-S100β (rabbit polyclonal)	Abcam	ab52642	1:500
Antibody	Anti-BrdU (rat monoclonal gG2a)	Abcam	ab6326	1:300
Antibody	Anti-Sox2 (rabbit polyclonal)	MilliporeSigma	AB5603	1:200
Antibody	Anti-PCNA (mouse monoclonal IgG2a)	MilliporeSigma	p8825	1:500
Antibody	Anti-active Caspase-3 (rabbit polyclonal)	BD Biosciences	559565	1:500
Antibody	Anti-HuC/D (mouse monoclonal IgG2b)	ThermoFisher	A-21271	1:300
Antibody	Anti-rabbit IgG-Alexa Fluor 546 (goal polyclonal)	Invitrogen	A11010	1:1000
Antibody	Anti-mouse IgG-Alexa Fluor 546 (goat polyclonal)	Invitrogen	A21133	1:1000
Antibody	Anti-mouse IgG2a-Alexa Fluor 647 (goat polyclonal)	Invitrogen	A21241	1:1000
Antibody	Anti-rat IgG Alexa Fluor 546 (goat polyclonal)	Invitrogen	A11081	1:500
Antibody	Anti-mouse IgG-Alexa Fluor 546 (goat polyclonal)	Invitrogen	A21143	1:1000
Chemical compound, drug	Cya	Toronto Chemical	C988400	10–100 μm
Chemical compound, drug	BMS-833923	Cayman Chemical (Akare et al., 2014)	16240	0.5–5 μm
Commercial assay or kit	Click-iT EdU imaging kit	Invitrogen	C10340	
Commercial assay or kit	DIG RNA labeling kit	Roche	11093657910	
Software, algorithm	GraphPad Prism	Graphpad Software	www.graphpad.com	
Software, algorithm	FIJI	PMID: 22743772	http://fiji.sc/	

**Table 2:** Genotyping primers for Hh Tet-On system

Transgenic line	Amplicon	Primers
*Tg(GBS-ptch2:RTTA-HA)^umz39Tg^*	RTTA-HA	Fw 5′-GAATTCACCATGTCTAGACTGGACA-3′
		Rv 5′-CTAACTGTCGACAGCGTAATCTGG-3′
*Tg(biTETRE:gli1,NLS-mCherry)^umz42Tg^*	mCherry	Fw 5′-CCAAGCTGAAGGTGACCAAG-3′
		Rv 5′-CTTGTAGATGAACTCGCCGTC-3′
*Tg(biTETRE:gli2aDR,NLS-mCherry)^umz43Tg^*	mCherry	Fw 5′-CCAAGCTGAAGGTGACCAAG-3′
		Rv 5′-CTTGTAGATGAACTCGCCGTC-3′

### Tissue preparation, sectioning, imaging, and cell counts

Larval and adult zebrafish were anesthetized using tricaine methanesulfonate (Sigma-Aldrich), and fixed overnight in 4% paraformaldehyde (PFA) at 4°C. For tissue sectioning, samples were washed three times in PBS/0.1% Tween (PBST), placed in embedding media (1.5% agar, 5% sucrose), incubated in 30% sucrose solution overnight at 4°C, and 20-μm sections were cut using a Leica CM 1950 cryostat. Larval brains were dissected after fixation and mounted in 25% glycerol. Imaging was performed using a Zeiss Axioskop 2 Apotome or LSM 700 laser scanning confocal microscope. Images are single optical planes except where noted. For lightsheet imaging, adult brains were fixed overnight in the cranium, dissected, embedded in acrylamide gel, and cleared as in Isogai et al. (2017). Lightsheet images were collected as optical sectioned z-stacks using a Zeiss Lightsheet Z1. Cell numbers and fluorescent intensity were quantified manually using ImageJ software (NIH) on single optical sections. Cells were outlined and the mean fluorescent intensity for each cell was calculated by drawing a circle of defined area through the brightest plane containing the nucleus.

#### Photoconversion of Kaede protein in Hh-responsive cells

Five- to 7-days post fertilization (dpf) *TgBAC(ptch2:Kaede)* transgenic larvae were anesthetized in MS-222 and mounted laterally or ventral-up in methylcellulose in a Vaseline well on a microscope slide. The jaw (brain conversion) or anterior trunk (spinal cord conversion) was centered under a 10× objective on a Zeiss Axioplan microscope and was UV irradiated for 10 min using the DAPI filter set (excitation 365 nm). For spinal cord analyses, the border between converted and unconverted Kaede protein was immediately photographed to ensure complete photoconversion. For brain analyses UV-irradiated fish were anesthetized and fixed overnight, followed by brain dissection and imaging to confirm complete photoconversion. For timed experiments, fish were removed from the microscope slide following photoconversion and placed in individual wells of a 12-well culture dish with or without small molecule inhibitors. At the indicated time points larvae were fixed for tissue processing (brain conversions) or were anesthetized and remounted for live imaging of the border between converted and unconverted Kaede.

### Cyclopamine (Cya) and BMS-833923 treatments

Three- to 7-d-old larvae were exposed to 10–100 μm Cya (Toronto Chemical; Incardona et al., 1998) by adding 10 μl of 10 mm stock solution (dissolved in DMSO) to 1 ml of embryo rearing medium (Westerfield, 2007) or fish system water in 12 well tissue culture plates (∼10 larvae/well) at 28.5°C. BMS-833923 (Cayman Chemical) was dissolved in 100% DMSO to make a 25 mm stock solution and fish were treated in 0.5–5 μm in fish system water. Control larvae were treated with 0.5% DMSO. Adults were injected intraperitoneally with 25 μl of 0.2 mg/ml Cya in DMSO (Reimer et al., 2009). Control animals were injected with DMSO.

#### Conditional gene activation using the Tet-On system

Progeny derived from adults carrying an RTTA-encoding driver transgene *(Tg(GBS-ptch2:RTTA-HA))* or *Tg(actb2:RTTA-HA;myl7:EGFP)* and a TRE-containing effector transgene *(Tg(TETRE:shha-mCherry; myl7:EGFP), Tg(biTETRE:gli1,NLS-mCherry) or Tg(biTETRE:gli2aDR,NLS-mCherry)* were sorted by GFP expression in the heart (*myl7:EGFP* transgene containing lines). RTTA-mediated gene expression was activated in 3- to 7-dpf larvae by adding 50 μg/ml (final concentration) doxycycline to system water (commonly used embryo media contained calcium and magnesium and can cause precipitation of doxycycline). Doxycycline-containing water was replaced every 12 h. Double transgenic embryos were identified by the presence of mCherry fluorescence using a Leica fluorescent dissecting microscope. Non-mCherry-expressing siblings served as controls, and could either carry one of the two parental transgenes (single-transgenic) or neither transgene (non-transgenic).

### Bromodeoxyuridine (BrdU) and ethynyldeoxyuridine (EdU) labeling

Three- to 7-d-old larvae were bathed in 10 mm BrdU or 3.3 mm EdU for 1–3 h, while adults were bathed for 2 d in 10 mm BrdU dissolved in 0.5% DMSO. Stocks solutions were 100 and 33.3 mm, respectively. Following fixation in 4% PFA BrdU was detected in tissue sections using the rat anti-BrdU (Abcam) or mouse anti-BrdU G3G4 (Developmental Studies Hybridoma Bank) antibodies at dilutions of 1:300 and 1:10, respectively. EdU was detected in fixed, dissected larval brains as described in the Click-It EdU Labeling kit (Invitrogen).

### Immunohistochemistry and *in situ* hybridization

Whole-mount larval immunohistochemistry was performed as in (Guner and Karlstrom, 2007) with a few changes. For anti-proliferating cell nuclear antigen (PCNA) labeling, dissected whole larval brains were incubated in 1× Histo-VT One (Nacalai Tesque) for 60 min at 65°C as in (Than-Trong et al., 2018). For anti-serotonin labeling dissected brains were digested in 60 μg/ml proteinase K for 30 min at room temperature before proceeding for whole mount immunohistochemistry. Immunohistochemistry on sectioned larval and adult tissue was performed as in Barresi et al. (2005). Primary antibodies are listed in the key resource table. Secondary Alexa Fluor-conjugated probes were purchased from The Jackson Laboratory and used at a concentration of 1:1000. *In situ* hybridization was performed as described previously (Shen et al., 2013) with probes to *notch1a* (Raymond et al., 2006), *erm* (Raible and Brand, 2001), *crabp1a* (Liu et al., 2005), *shha* (Krauss et al., 1993), *ptch2* (Concordet et al., 1996), and *gli1*, *gli2a*, and *gli3* (Devine et al., 2009).

### Statistical analyses

Statistical analyses were done using Prism GraphPad software. For experiments involving two groups, treatment groups were compared using a Student’s *t* test. A one-way ANOVA with Tukey’s multiple comparison was used to determine statistical significance for multiple comparisons. Sample size was not predetermined by statistical methods, but was based on similar studies in the field. For all experiments animals from the same clutch were randomly assigned to experimental conditions before manipulations, with adult animals also being chosen based on similarity in standard length at the beginning of the experiment. Cell numbers are represented as mean ± SD, with statistical significance indicated in figures as *p ≤ 0.05, **p < 0.01, ***p < 0.001, ****p < 0.0001.

## Results

### Hh-responsive cells in the adult hypothalamus are proliferative neural precursors

**Figure 1.**
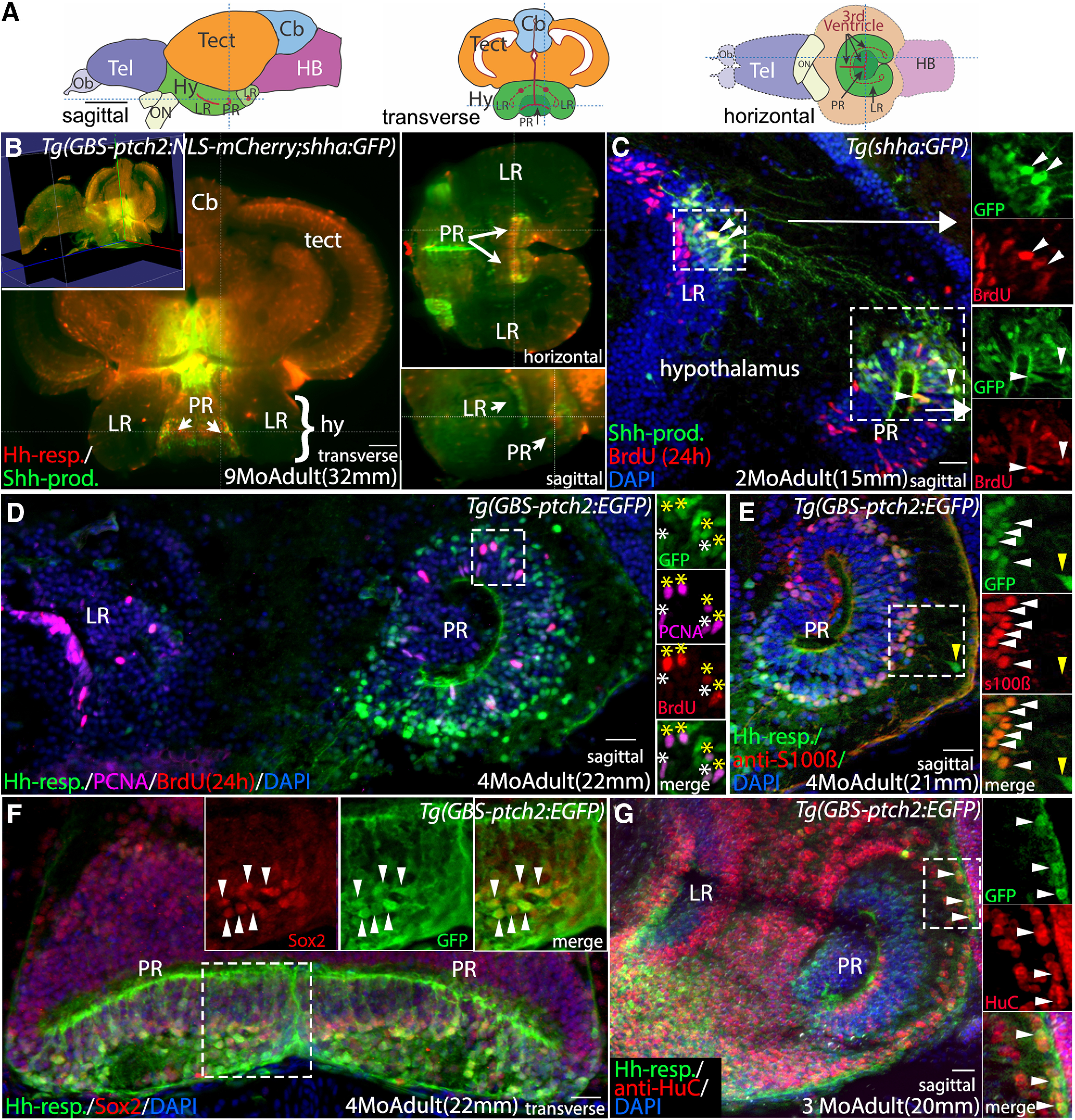
Shh-producing and Hh-responsive radial glial cells in the adult hypothalamus are proliferative neural precursors. ***A***, Schematic sagittal, transverse, and horizontal sections through the adult zebrafish brain. ***B***, Optical sections from a whole-brain light-sheet confocal image of a *Tg(GBS-ptch2:NLS-mCherry;shha:GFP)* double transgenic adult showing Hh-responsive cells (Hh-resp, red) in relation to Shh-producing cells (Shh-Prod, green) in the LR and PR of the adult hypothalamic (third) ventricle. Shh-expressing cells are adjacent to Hh-responsive cells in the ventricular zone of both recesses. Yellow lines in each panel indicate the plane of section in the other panels. Video 1 shows a progression through optical sections (from posterior to anterior), and Video 2 shows a 3-dimensional rotating view of this image. ***C***, Sagittal tissue section showing Shh-producing cells (green) and BrdU-labeled proliferative cells (red, 24-h exposure) in the adult hypothalamus. Shh-producing cells are located primarily in the dorsal portions of both the LR and PR. Shh-expressing cells in the LR send projections toward to the dorsal region of the PR. Panels at right show separated fluorescent channels from the boxed regions, with examples of co-labeled cells in both ventricular regions indicated by arrowheads. ***D***, Sagittal tissue section through the LR and PR of a *Tg(GBS-ptch2:EGFP)* transgenic adult showing Hh-responsive cells and proliferative cells labeled with BrdU (red, 24-h treatment) and the anti-PCNA antibody (magenta). Panels at right show separated channels from the boxed region, with four PCNA^+^/BrdU^+^-labeled Hh-responsive cells indicated by yellow asterisks and two PCNA^+^/BrdU^–^ cells indicated by white asterisks. ***E***, Sagittal tissue section through the PR of a *Tg(GBS-ptch2:EGFP)* transgenic adult labeled with the anti-s100β antibody to show radial glia. Most but not all Hh-responsive cells are s100β-positive (white arrowheads). A small percentage of GFP-labeled cells that are more distant from the ventricle are s100β-negative (yellow arrowhead), suggesting these cells have differentiated but retain GFP fluorescence from previous GBS-*ptch2:EGFP* transgene expression. ***F***, Transverse tissue section through the PR of the hypothalamus of a *Tg(GBS-ptch2:EGFP)* adult labeled with the Sox2 antibody that labels neurogenic cells. Most or all Hh-responsive cells express the Sox2 protein. Panels at right show separated channels from the boxed region with arrowheads marking co-labeled cells. ***G***, Sagittal tissue section through the PR of a *Tg(GBS-ptch2:EGFP)* adult labeled with an antibody to the neuronal marker HuC/D (now called Elavl3). Double labeling of cells far from the ventricle indicates that Hh-responsive cells (green) can give rise to HuC/D-expressing neurons (red). Panels at right show separated channels from the boxed region with co-labeled cells (arrowheads). Cb, cerebellum; hy, hypothalamus; LR, lateral recess of the hypothalamic (third) ventricle; PR, posterior recess of the hypothalamic (third) ventricle; tect, tectum. Scale bars: 1 mm (***A***), 50 μm (***B***), and 20 μm (***C–H***).

**Figure 2.**
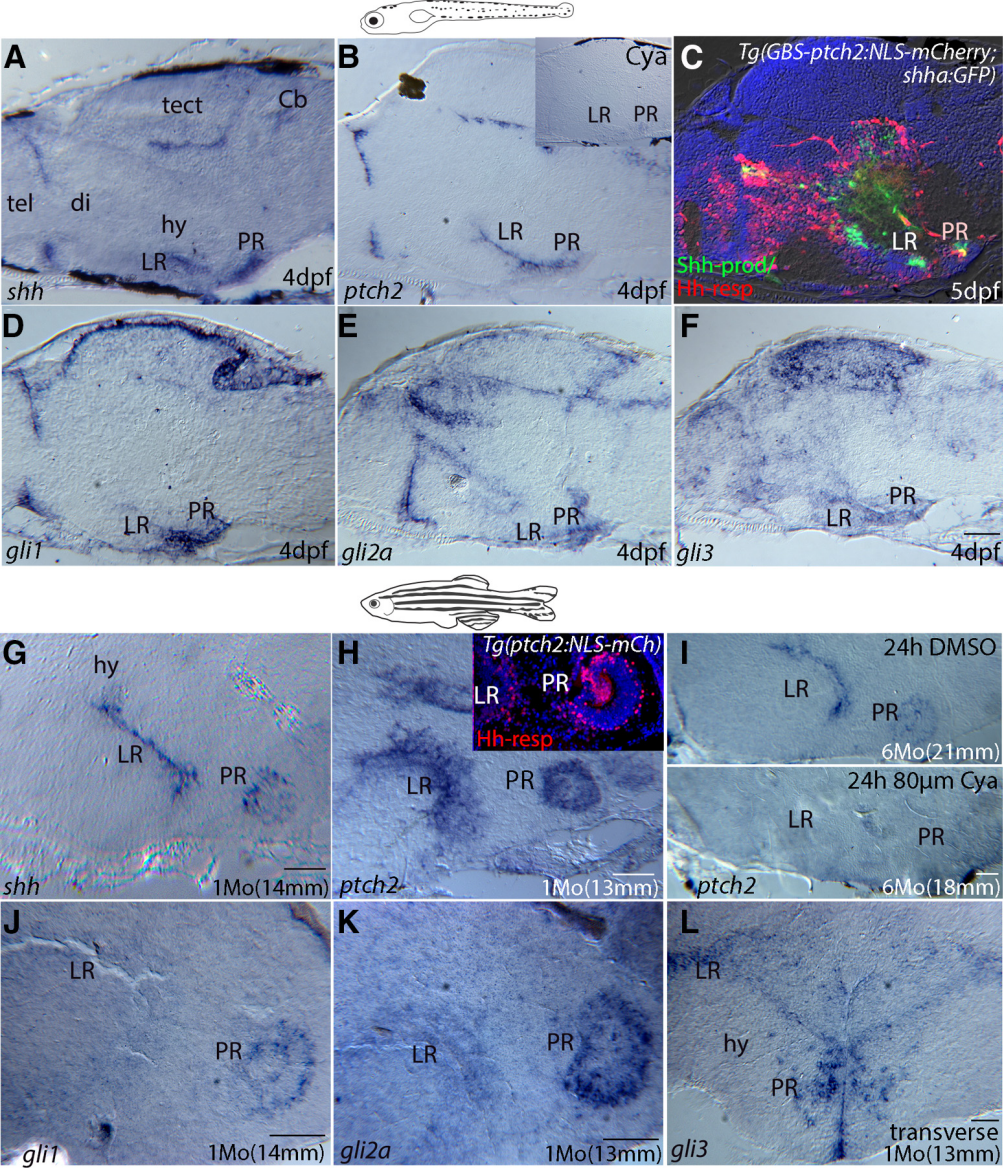
Hh pathway gene expression in the larval and adult zebrafish hypothalamus. ***A–F***, Expression of Hh-signaling pathway genes in sectioned tissue from 4-dpf larvae. ***A***, *In situ* hybridization (ISH) showing that *shh* is highly expressed in the ventricular regions of the LR and PR of the hypothalamic ventricle, as well as in ventricular regions of the diencephalon/telencephalon border, cerebellum, and tectum. ***B***, The Hh-target gene *patched2* is similarly expressed in ventricular regions throughout the larval brain, with *ptch2* transcription being eliminated by treatment with Cya (***B***, inset). ***C***, Shh-producing (green, Shh-prod) and *ptch2* (red, Hh-resp) expression in the larval brain as revealed by the *Tg(shha:GFP)* and *Tg(GBS-ptch2:NLS-mCherry)* reporter lines, seen here in a double transgenic larva. This midline section reveals Hh response in the midline ventricular region**. *D–F***, ISH showing expression of the Hh-responsive transcription factors *gli1*, *gli2a*, and *gli3*, respectively. ***G–L***, ISH on tissue sections showing expression of Hh signaling pathway genes in the adult hypothalamus. ***G***, *shh* expression is maintained in the LR and PR of the adult hypothalamic ventricle. ***H***, Expression of the Hh-target gene *patched2* in the hypothalamic ventricular regions as revealed by ISH and compared with nuclear mCherry expression in cells of the LR and PR driven by the *ptch2* promoter construct in the *Tg(GBS-ptch2:NLS-mCherry)* transgenic line (inset). ***I***, ISH using a *ptch2* probe shows that Cya treatment (bottom panel) of six-month-old adult dramatically reduced *ptch2* gene expression in the LR and PR compared with an age-matched DMSO control treated fish (top panel). ***J–L***, ISH showing expression of the Hh-responsive transcription factors *gli1*, *gli2a*, and *gli3*, respectively, in the hypothalamus. All panels show sagittal tissue sections, except ***L***, which shows a transverse tissue section. cb, cerebellum; di, diencephalon; hy, hypothalamus; tect, tectum; tel, telencephalon. Scale bars: 50 μm.

We first took advantage of zebrafish transgenic reporter lines and new light-sheet imaging techniques to characterize the cellular Hh response in the adult hypothalamus (Fig. 1). As in other vertebrate brain regions, proliferation in the adult hypothalamus occurs in the ventricular regions. In zebrafish the hypothalamic ventricle includes two “recesses” that extend laterally from the midline ventricle (Wullimann et al., 1996; Fig. 1*A*). The lateral recess (LR) is anterior and largely lateral to the posterior recess (PR). Using modified CLARITY tissue clearing protocols (Isogai et al., 2017) and whole-brain light sheet microscopy we documented the relationship of Hh-responsive and sonic Hh (Shh)-producing cells in adults carrying the *GBS-ptch2:NLS-mCherry* and *shha:GFP* transgenes (Neumann and Nuesslein-Volhard, 2000; Shen et al., 2013; Fig. 1). Hh signaling in the ventricular regions is accurately reflected in the *ptch2* and *shha* reporter lines as verified by *in situ* labeling of cells expressing *shh*, *ptch2*, as well as the Hh-pathway transcription factors *gli1*, *gli2a*, and *gli3* (Figs. 1, 2). Whole-brain light sheet confocal imaging revealed that Hh-responsive cells reside adjacent to Shh-producing cells in the PR and LR of the hypothalamic ventricle (Fig. 1*B*; Movie 1, Movie 2). Analyses of sectioned adult brain tissue showed that Shh-producing cells have radial glial morphology within the LR and PR, and that they are proliferative, as indicated by BrdU labeling (Fig. 1*C*). A subset of Shh-producing cells in the LR extend processes to the PR (Fig. 1*C*).

Movie 1. Hh signaling in the adult zebrafish brain as revealed by whole brain light sheet imaging: transverse optical sections. Video of transverse optical sections through the nine-month-old *Tg(GBS-ptch2:NLS-mCherry;shha:GFP)* double transgenic adult brain shown in Figure 1*B*, progressing from posterior (hindbrain) to anterior (telencephalon). Shh-producing cells are visualized in green and Hh-responsive cells are seen in red. The PR of the hypothalamus is visible in the ventral brain from *t* = 35 s to *t* = 40 s in the video. 10.1523/ENEURO.0226-20.2020.video.1

Movie 2. Hh signaling in the adult brain as revealed by whole-brain light sheet imaging: 3-dimensional rotation. 3-Dimensional rotating view of the nine-month-old *Tg(GBS-ptch2:NLS-mCherry;shha:GFP)* adult brain shown in Figure 1*B*. Shh-producing cells are visualized in green and Hh-responsive cells are seen in red. 10.1523/ENEURO.0226-20.2020.video.2

We focused our further analysis on the Hh-responsive cells to better characterize this population and understand how Hh signaling affects neurogenesis in the hypothalamus. BrdU and PCNA-labeling of adult fish carrying the *ptch2:GFP* and *ptch2:NLS-mCherry* transgenes revealed that Hh-responsive cells are primarily located in the PR (Fig. 1*D*). These cells appear to be radial glia based on their morphology, with processes that span the distance from the pial (basal) surface of the brain to the ventricle (Fig. 1*D*,*E*,*G*). Approximately 12% of Hh-responsive cells in the adult PR are proliferative, as revealed by expression of the PCNA and by BrdU incorporation (Fig. 1*D*, see Fig. 7). A majority of Hh-responsive cells in the PR express the glial marker s100β (Fig. 1*E*) and all Hh-responsive cells examined appear to express the neural precursor transcription factor Sox2 (Fig. 1*F*). These cells appear to give rise to neurons, as shown by labeling with an antibody to the neuronal protein HuC (Park et al., 2000; Fig. 1*G*). Together, these data demonstrate that a subset of radial glial cells in the PR of the hypothalamic ventricle are responsive to Hh signaling in adults, and that these cells are both proliferative and neurogenic. Thus, these cells represent a previously undescribed population of neural precursors in the adult vertebrate hypothalamus.

### Hh signaling positively regulates hypothalamic precursor proliferation

**Figure 3.**
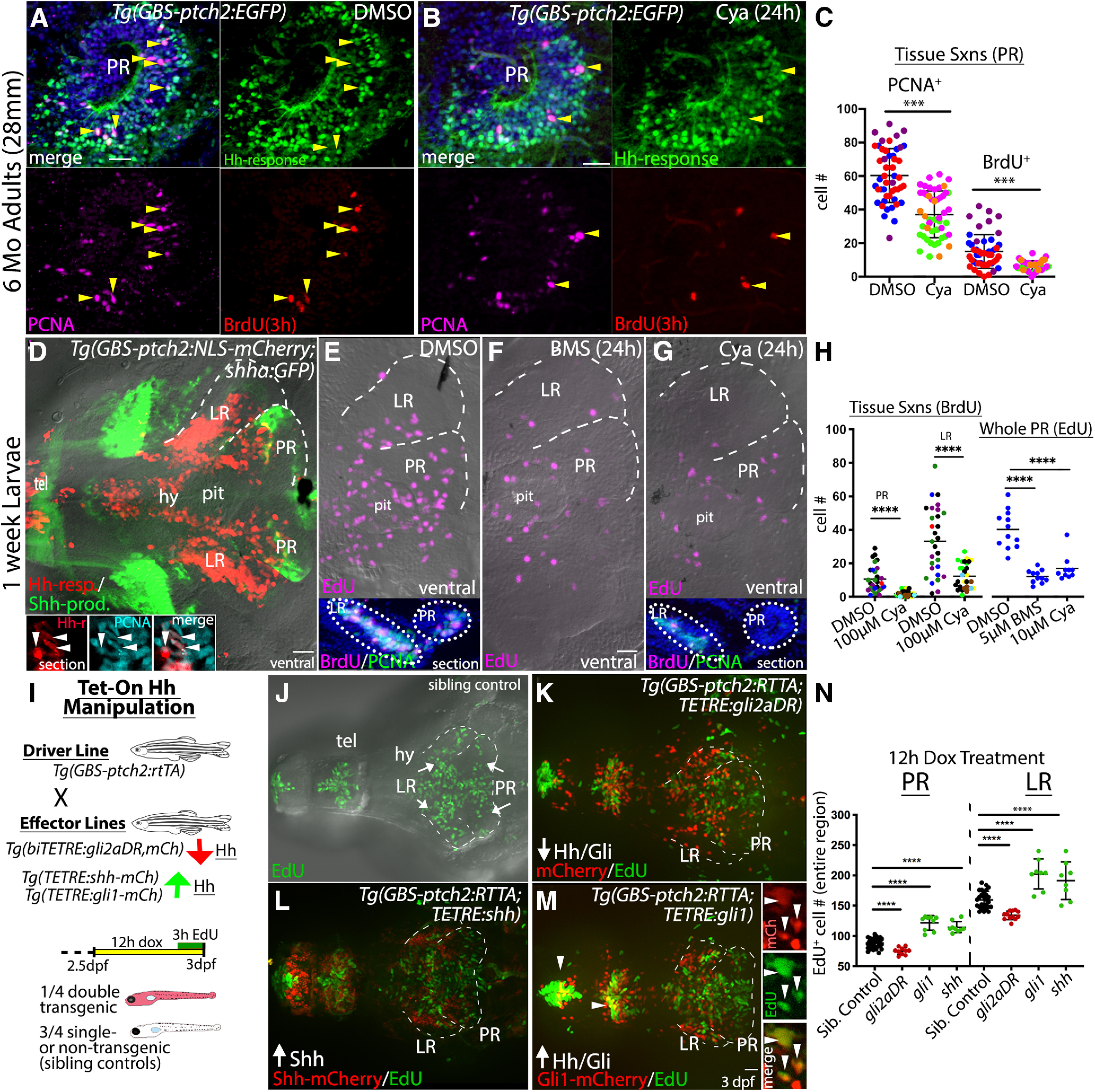
Hh signaling positively regulates proliferation in the adult and larval hypothalamus. ***A***, Sagittal section through the PR of a DMSO (control)-injected *Tg(GBS-ptch2:EGFP)* transgenic adult brain showing anti-PCNA (magenta)-labeled and BrdU (red, 3-h exposure)-labeled proliferative cells. Arrowheads mark triple-labeled, proliferative, Hh-responsive cells. ***B***, Sagittal section through the PR of a *Tg(GBS-ptch2:EGFP)* transgenic adult brain 24 h after injection of the Hh/Smo inhibitor Cya (80 μm) showing anti-PCNA (magenta)-labeled and BrdU (red)-labeled proliferative cells. Arrowheads mark triple-labeled cells. ***C***, Quantification of BrdU-labeled and PCNA-labeled cells in control and Cya-injected adults. Each dot represents the number of cells counted in a single tissue section, with each color representing a different adult fish (*n* = 3 fish, 10–11 sections per fish). ***D***, Ventral view of a dissected *Tg(GBS-ptch2:NLS-mCherry;shha:GFP)* double transgenic larval brain showing Hh-responsive (red) and Shh-producing (green) cells. The hypothalamic lobes surrounding the PR and LR of the third ventricle are outlined on one side of the brain. Insets show PCNA-labeled (cyan) Hh-responsive cells (red) in sagittal sections through the PR of a different larval brain, with examples of double-labeled cells marked by arrowheads. ***E–G***, Ventral views of 7-dpf larval brains from fish labeled for 3 h with EdU following incubation in DMSO (control, ***E***), BMS-833923 (BMS, ***F***), or Cya (***G***) for 24 h. Insets in ***E***, ***G*** show representative sagittal tissue sections through the hypothalamus of DMSO (***E***)-treated and Cya (***F***)-treated larvae labeled to show BrdU (2-h treatment) and PCNA-labeled proliferative cells. ***H***, Quantification of EdU-labeled, BrdU-labeled, and PCNA-labeled cells in the PR and LR. For tissue sections (left), each color represents a different larval fish (*n* = 5 controls, *n* = 6 Cya treated), and each dot represents cells in a single tissue section (five to nine sections per fish). For whole PR EdU counts (right), each dot represents one larva (*n* = 12 for DMSO, 11 for BMS, and 10 for Cya). ***I***, Schematic showing the Tet-On transgenic system used to manipulate Hh signaling. The *Tg(GBS-ptch2:RTTA)* line drives expression of the RTTA transcriptional activator in Hh-responsive cells, with different effector transgenes allowing upregulation and downregulation of Hh signaling on the addition of doxycycline, which is required for RTTA function. Activation of the two-part transgenic expression system is indicated by mCherry fluorescence in larvae, as shown in the diagram of the experimental timeline. ***J–M***, Ventral views of EdU-labeled (3-h exposure) proliferative cells in the larval hypothalamus following manipulation of Hh signaling levels using the Tet-On system. ***J***, EdU-labeled proliferative cells (green) in a single-transgenic sibling (control), identified based on the lack of red fluorescence. ***K***, EdU-labeled proliferative cells (green) visualized 12 h after activation of a dominant repressor form of the Gli2 transcription factor (Gli2DR) in Hh-responsive cells (red). ***L***, EdU-labeled proliferative cells (green) visualized following activation of the Shh effector transgene [*Tg(TETRE:shha-mCherry)*] in Hh-responsive cells [*Tg(GBS-ptch2:RTTA)* driver]. ***M***, EdU-labeled proliferative cells 12 h after activation of the Gli1-mCherry fusion protein in Hh-responsive cells (red). A subset of EdU-labeled cells was co-labeled with the mCherry protein (arrowheads). Separated EdU, mCherry, and merged channels from the PR of a different brain are shown on the right. ***N***, Quantification of all EdU^+^ proliferating cells in the PR and LR of the larval hypothalamus following Hh-manipulation using the Tet-On system. Each dot represents an individual fish. Upregulation of Hh/Gli signaling via the *shh*a and *gli1* transgenes led to increased proliferation, while downregulation of Hh signaling via the *gli2DR* transgene reduced proliferation; ***p < 0.001, ****p < 0.0001. Source data for graphs can be found in Extended Data Figure 3-1. Scale bars: 20 μm.

To determine whether Hh signaling affects adult neurogenesis we injected adult fish with the Hh inhibitor Cya, which blocks Hh signaling at the level of the smoothened (Smo) protein (Incardona et al., 1998). Intraperitoneal injections of Cya reduced proliferation throughout the hypothalamus 24 h later as assayed using BrdU incorporation and PCNA labeling (Fig. 3*A–C*). Intraperitoneal injection of Cya also dramatically reduced *ptch2* transcription as assayed by *in situ* hybridization, confirming a loss of Hh signaling in the hypothalamic ventricular regions (Fig. 2*I*). GFP fluorescence was still visible in Hh-responsive cells 24 h after Cya injection because of the perdurance of the GFP fluorescent reporter protein (Fig. 3*A*,*B*). Quantification of BrdU-labeled and PCNA-labeled cells in the PR revealed an ∼50% reduction in hypothalamic proliferation (Fig. 3*C*; Extended Data Fig. 3-1).

10.1523/ENEURO.0226-20.2020.f3-1 Extended Data Figure 3-1 Source data for graphs in Figure 3. Spreadsheet with PCNA+, BrdU+, and EdU+ cell quantification data used to generate graphs in Figure 3, with exact *p* values. Download Figure 3-1, XLSX file.

**Figure 4.**
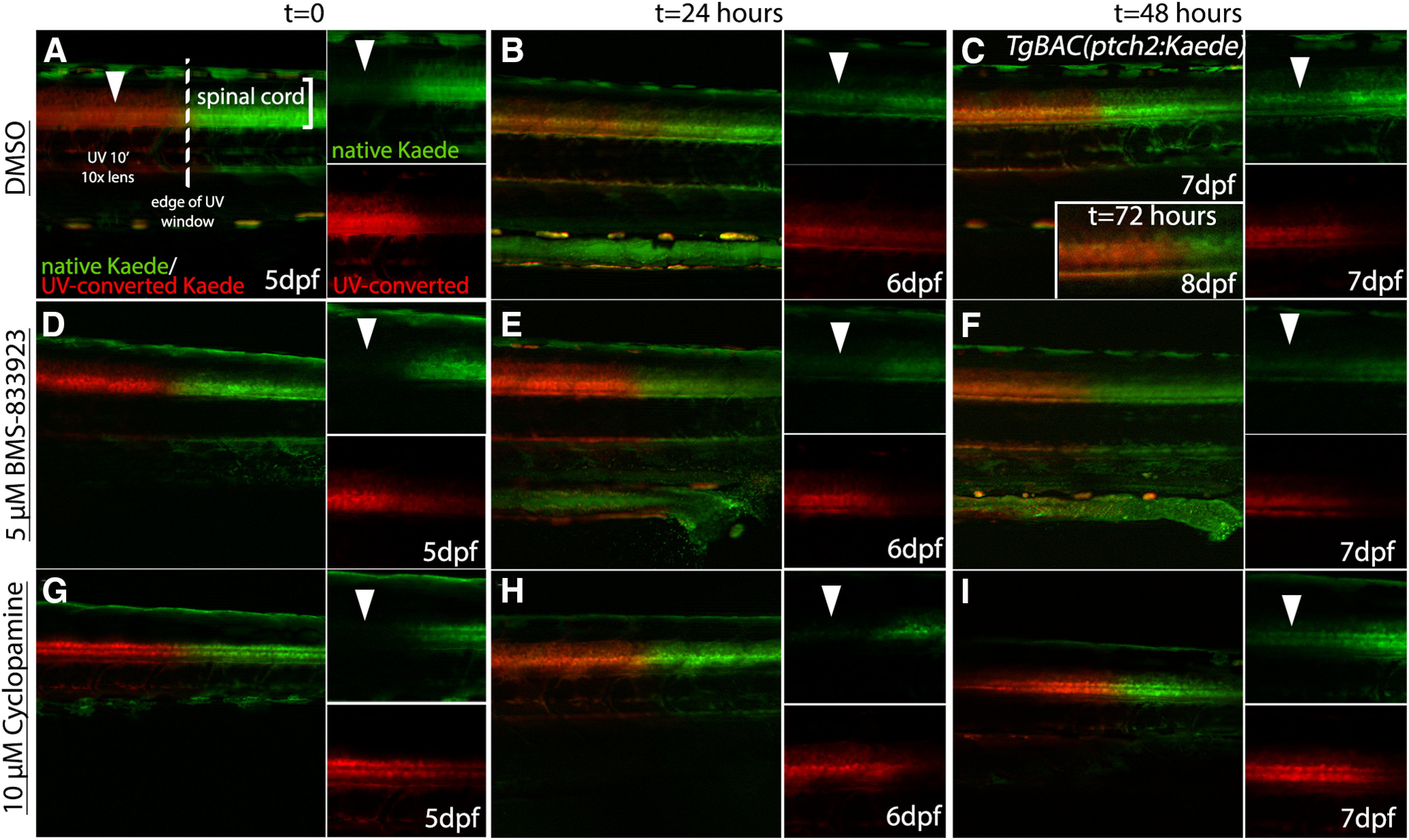
Cya and BMS-833923 both block Hh signaling in zebrafish larvae. ***A–I***, Lateral views of the trunk of *TgBAC(ptch2:Kaede)* larvae showing Kaede expression in Hh-responsive cells of the ventral spinal cord at different time points after UV photoconversion. Smaller panels show separated color channels. ***A***, ***D***, ***G***, The border of native (green) and photoconverted (red) Kaede protein in the spinal cord immediately after photoconversion of the anterior trunk region. ***B***, ***C***, In DMSO-treated control larvae newly synthesized Kaede protein (arrowheads) was easily detectable after 24 h and continued to increase through 48 and 72 h, when converted and non-converted protein levels were very roughly equivalent (***C***, inset). ***E***, ***F***, Treatment with 5 μm BMS-833923 effectively blocked the synthesis of new Kaede protein (arrowheads) in the ventral spinal cord at 24 h (***E***), with low levels of newly synthesized Kaede protein becoming visible after 48 h at this drug concentration (***F***). ***H***, ***I***, Treatment with 10 μm Cya effectively blocked new Kaede synthesis (arrowheads) for 24 h (***H***), with low levels of expression being visible after 48 h at this low Cya concentration (***I***).

We next turned to larval stages to facilitate Hh signaling gain-of-function and loss-of-function analyses. As in adults, Shh-producing and Hh-responsive cells were distributed throughout the larval hypothalamic ventricular zones, with Hh-responsive cells being much more prevalent in the LR (Fig. 3*D*). A substantial subset of Hh-responsive cells was seen to be proliferative as revealed by PCNA labeling (Fig. 3*D*, inset). Treating 6-dpf larvae for 24 h with Cya, or with the more specific Smo inhibitor BMS-833923 (Akare et al., 2014; Armstrong et al., 2017), reduced proliferation on both the LR and PR by ∼75% (Fig. 3*E–H*; Extended Data Fig. 3-1). The effectiveness of these molecules in blocking Hh signaling was verified using the *ptch2:Kaede* line (Huang et al., 2012), which expresses the photoconvertible Kaede protein in Hh-responsive cells (Fig. 4). Both BMS-833923 and Cya effectively blocked new Kaede transgene expression during the treatment period, and both drugs had similar effects on hypothalamic proliferation. These data strongly suggest that Hh signaling affects proliferation through the Smo-mediated Hh signaling pathway.

**Figure 5.**
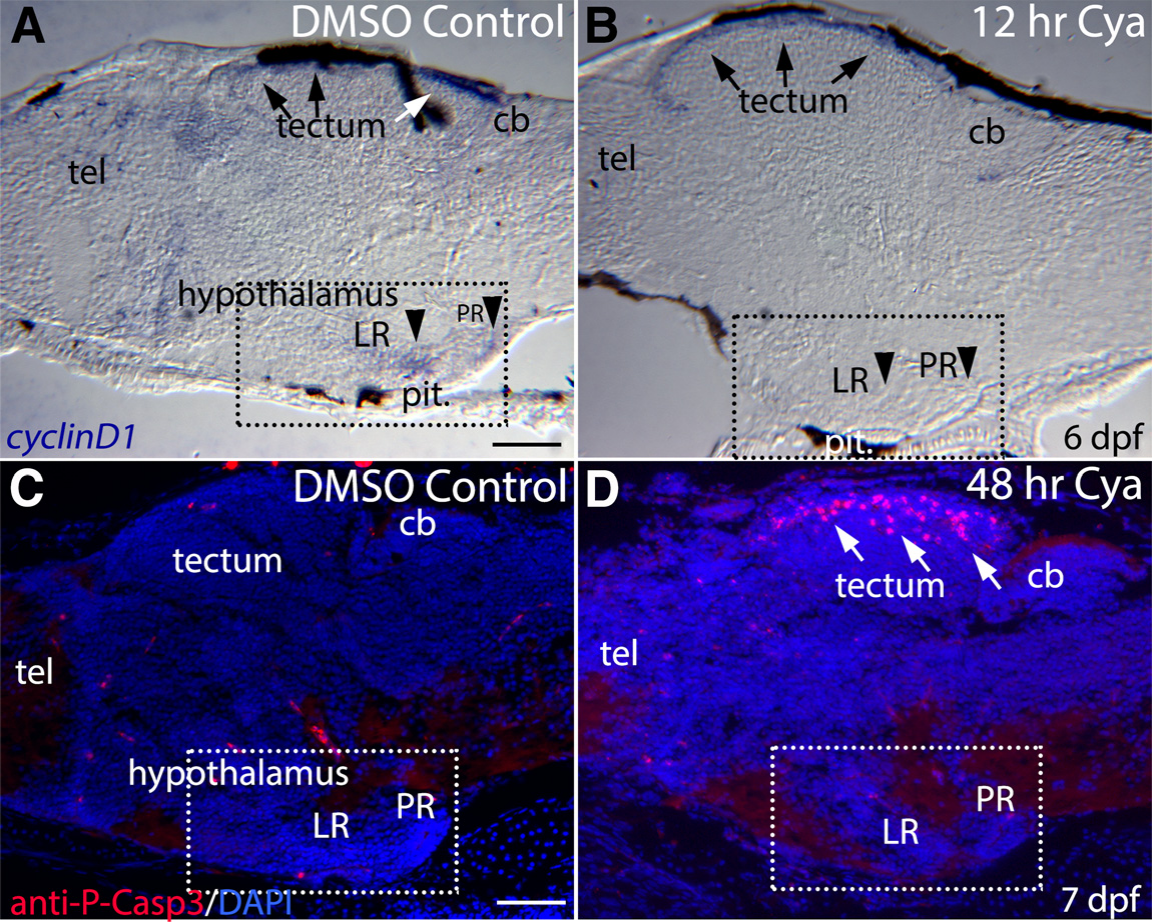
Blocking Hh signaling with Cya reduces *cyclinD1* expression in the hypothalamus but does not increase hypothalamic cell death. ***A***, Sagittal section through a 6-dpf larval brain showing *cyclinD1* mRNA expression in the ventricular regions of the hypothalamus (dashed-square), telencephalon (tel), and dorsal tectum (arrows). ***B***, *cyclinD1* is expression is eliminated in the LR and PR of the hypothalamus (arrowheads) following 12 h of Cya exposure but is largely unaffected in the tectum (arrows). ***C***, Cell death in a DMSO-treated control larva as revealed by anti-phospho-Caspase3 antibody labeling. ***D***, Two-day Cya treatment did not lead to increased cell death in the hypothalamus, although increased cell death was seen in the tectum (arrows). cb, cerebellum; tect, tectum; tel, telencephalon. Scale bars: 50 μm.

Relatively short (12 h) Cya exposure eliminated *cyclinD1* mRNA in the ventral brain but did not affect more dorsal expression of this cell-cycle gene (Fig. 5*A*,*B*), consistent with Hh signaling acting on hypothalamic proliferation by regulating cell cycle progression. We saw no evidence of increased cell death in the hypothalamus of Cya-treated larvae, as assayed using an antibody against activated-Caspase3, although we did observe an intriguing increase in cell death in the dorsal tectum (Fig. 5*C*,*D*).

To further verify the specificity of the effects seen using these small molecule Hh/Smo inhibitors, we next used the two-part Tet-On conditional gene regulation system (Campbell et al., 2012). This two-part system provides spatiotemporal control of Hh signaling and allows manipulation of Hh signaling at the level of the Gli transcription factors (Fig. 3*I*). A truncated Gli2a transcription factor (Gli2aDR) was shown to dominantly repress Hh signaling at the transcriptional level, while expression of Shh or the full-length Gli1 transcription factor activates Hh signaling (Karlstrom et al., 2003; Shen et al., 2013). Expression of the Gli2aDR protein in Hh-responsive cells resulted in a 25% reduction in hypothalamic proliferation across both the LR and PR within 12 h of transgene activation (Fig. 3*K*,*N*), with mCherry expression visible within 4–6 h of transgene activation (data not shown). Similarly, activation of a Gli2a dominant repressor transgene using the heat-shock inducible system (Shen et al., 2013) reduced proliferation by ∼50% (data not shown). We next upregulated Hh signaling by expressing either Shha or the full length Gli1 transcriptional activator in Hh-responsive cells. Expression of either Shha or Gli1 increased hypothalamic proliferation by 20–30% within 12 h of transgene activation (Fig. 3*L–N*; Extended Data Fig. 3-1). mCherry expression was seen in a subset of EdU-labeled cells ectopically expressing the Gli1 transcription factor (Fig. 3*M*), but not in cells expressing the secreted Shh ligand (Fig. 3*L*) or the Gli2DR transcriptional repressor (Fig. 3*K*), consistent with a cell-autonomous role for Gli-mediated Hh signaling in positively regulating precursor proliferation. Together, these data indicate that Hh signaling mediated by the Gli transcription factors is both necessary and sufficient for normal proliferation rates in the larval hypothalamus.

### The PR as a heterogeneous stem cell niche under the influence of multiple cell-signaling systems

**Figure 6.**
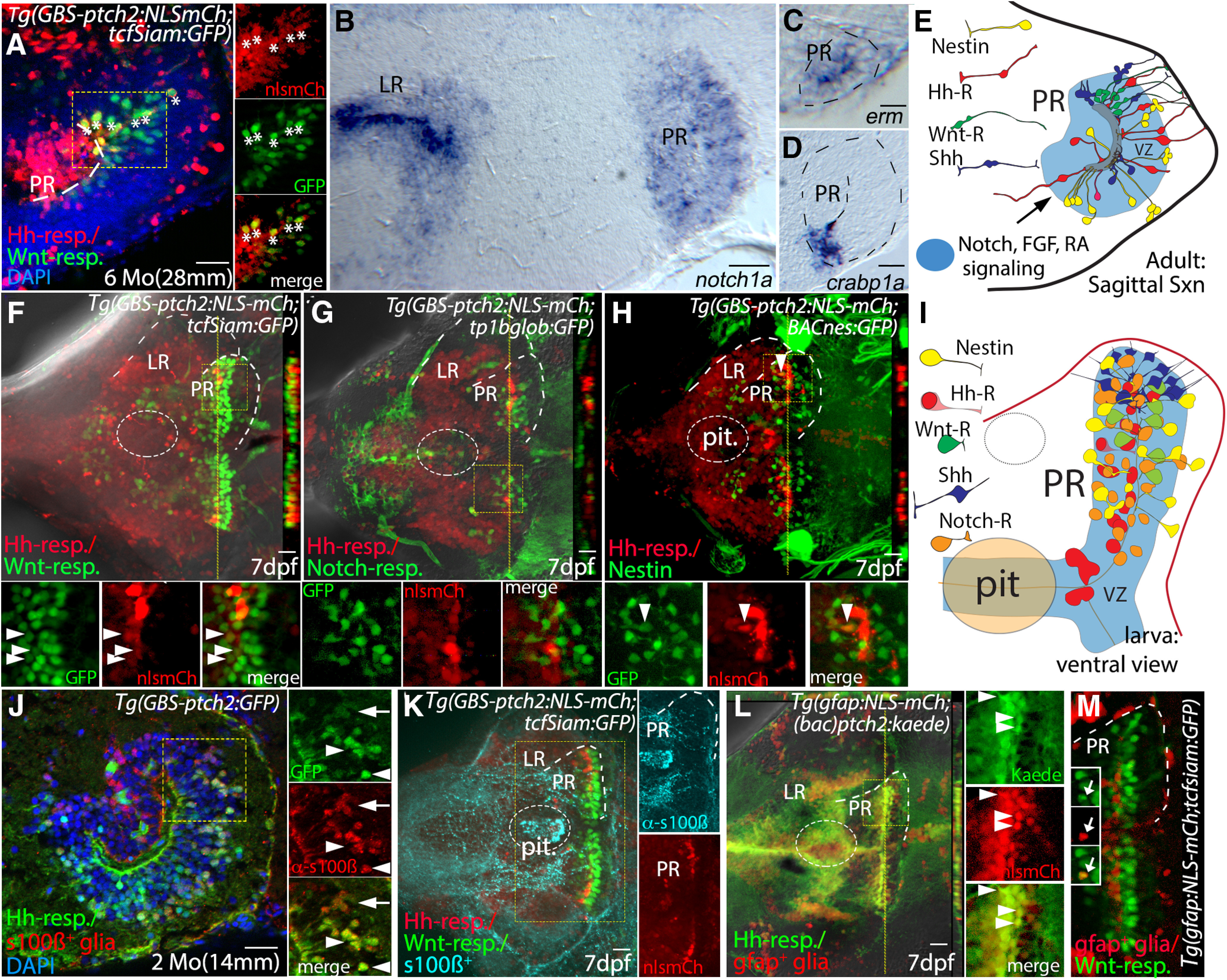
Hh, Wnt, Notch, FGF, and retinoic acid signaling in a complex hypothalamic neurogenic niche. ***A***, Sagittal section through the PR of a *Tg(GBS-ptch2:NLS-mCherry;TCFSiam:GFP)* double transgenic adult. Hh-responsive cells (red) of the PR are largely distinct from Wnt-responsive cells (green); however, a subset of cells in the dorsal PR contains both GFP and mCherry (asterisks). Panels at right show separated channels from the boxed region. ***B***, Sagittal section through the hypothalamus showing *notch1a* expression in the LR and PR, as visualized by *in situ* hybridization. ***C***, ***D***, Sagittal section through the PR showing expression of the FGF target gene *erm* (Raible and Brand, 2001) and the retinoic acid binding protein gene *crabp1a* (Liu et al., 2005). ***E***, Schematic of cell-cell signaling systems of the PR, including four distinct radial glial types that are defined by reporter gene expression (see Figs. 1-3 for data used to draw Shh-expressing and nestin-expressing cells). ***F–H***, Ventral views of the 7-dpf hypothalamus of double transgenic larvae. Dotted lines outline the ventricular regions of LR and PR, and cut views at right show optical *Z*-sections through the PR at the position of the yellow dotted line. ***F***, Larval Hh-responsive cells of the PR are distinct from Wnt-responsive cells, as revealed in *Tg(GBS-ptch2:NLS-mCherry;TCFSiam:GFP)* double transgenic larva. ***G***, Hh-responsive and Notch-responsive cells of the PR are also distinct, as revealed in *Tg(GBS-ptch2:NLS-mCherry;tp1bglob:GFP)* double transgenic larva. ***H***, Hh-responsive cells of the PR are distinct from nestin-expressing cells, as revealed in *Tg(GBS-ptch2:NLS-mCherry;nes:EGFP)* double transgenic larva. ***I***, Schematic ventral view of the larval hypothalamus showing cell-signaling pathways examined and four distinct radial glial types, as defined by gene expression in fluorescent reporter lines. Note that the data do not rule out the possibility that subsets of different signal-responsive cells may overlap both spatially and temporally. ***J***, Sagittal section through the PR of a *Tg(GBS-ptch2:GFP*) adult labeled with the anti-s100β antibody. A total of 482 of 633 (76%) s100β-expressing cells were Hh responsive based on double labeling (arrowheads), while 482 of 533 (90%) of Hh-responsive cells co-labeled with the s100β antibody (*n* = 6 sections from 2 brains). Weakly labeled S100β cells tended to correspond to the more weakly fluorescent GFP+ cells (arrows). ***K***, In 7-dpf larvae, S100β was only weakly expressed in the PR, despite strong expression in other regions of the brain and pituitary (ventral view). ***L***, Ventral view of a *Tg(gfap:NLS-mCherry;GBS-ptch2:kaede)* double transgenic larva. A total of 176 of 474 (37%) of *gfap*-expressing cells counted were Hh responsive based on Kaede expression (arrowheads). Conversely, 176 of 463 (35%) Hh-R cells counted expressed the *gfap* transgene (*n* = 10 larvae). ***M***, Ventral view of a *Tg(gfap:NLS-mCherry;tcfsiam:GFP)* double transgenic larva. Only 12 of 572 (2%) Wnt-R cells examined expressed the *gfap* transgene (insets, arrows; *n* = 10 embryos). pit, pituitary. Scale bars: 20 μm.

To explore potential interactions between Hh and other signaling pathways, we next determined the spatial relationship between Hh, Wnt, and Notch in the hypothalamic ventricular zone. Examination of adult brains from fish carrying both the Wnt- (*tcfsiam:GFP*) and Hh- (*ptch2:NLS-mCherry*) transcriptional reporters revealed that the Wnt and Hh signaling systems are active in overlapping but distinct regions of the PR. Wnt-responsive cells were positioned primarily in the dorsal PR while Hh-responsive cells were distributed throughout the ventricular zone (Fig. 6*A*). Closer examination of double transgenic adults revealed that a small subset of cells expressed both GFP and mCherry (Fig. 6*A*, insets), suggesting either simultaneous or sequential (given the perdurance of the mCherry and GFP proteins) activation of Wnt and Hh signaling in these cells.

Given the data indicating that Notch signaling acts to keep telencephalic neural progenitors in a quiescent state (Chapouton et al., 2007; Kaslin et al., 2009; Ganz et al., 2010), we next examined whether Notch signaling genes were expressed in the adult zebrafish hypothalamus. *In situ* hybridization using the *notch1a* (Fig. 6*B*) as well as *notch1b* and *deltaB* (data not shown) probes revealed ventricular expression of these genes in the LR and PR of the hypothalamic ventricle. Active Notch signaling in the larval PR was indicated by the expression of GFP in radial glia of fish carrying the *tp1:GFP* Notch-reporter transgene (Parsons et al., 2009; Fig. 6*G*). The FGF target gene *erm* (Fig. 6*C*), as well as the gene encoding the retinoic acid binding protein Crabp1a (Fig. 6*D*), are also expressed in the adult PR, suggesting these signaling systems may be involved in precursor regulation. A schematic summarizing the signaling environment of the adult PR defined by these expression analyses is shown in Figure 6*E*.

We next examined the spatial relationships of different progenitor populations in larvae by generated double-transgenic fish carrying the Wnt or Notch GFP reporter transgenes in combination with the *ptch2:NLS-mCherry* reporter that labels nuclei of Hh-responsive cells. These analyses revealed that Wnt-responsive and Hh-responsive cells in the PR comprise largely non-overlapping populations (Fig. 6*F*). We identified a small number of mCherry/GFP containing cells in larvae, as seen in adults (Fig. 6*F*, arrowheads). A relatively small number of Notch-responsive cells, as seen in the *tp1:GFP* Notch reporter line (Parsons et al., 2009), was distributed throughout the PR, and we failed to identify Notch-responsive cells that expressed the *ptch2:NLS-mCherry* reporter transgene (Fig. 6*G*). Finally, larval Nestin-expressing and Hh-responsive cells comprise largely distinct precursor populations in the PR, with a small number of co-expressing cells being seen near the ventricle (Fig. 6*H*, arrowheads). A schematic diagram summarizing these data and the signaling environment of the larval PR is presented in Figure 6*I*.

We next determined the proportion of radial glial cells that were Hh-responsive using the radial glial markers s100β or glial fibrilary acidic protein (GFAP; Fig. 6*J–L*). In two-month-old (14 mm) adults, about ¾ of S100β+ cells in the PR were Hh-responsive (482/633 cells counted; Fig. 6*J*). In turn, ∼90% of Hh-R cells expressed s100β (482/533 cells), consistent with the majority of these cells being proliferative radial glia. In general, cells that were weakly labeled with the anti-s100β antibody had lower levels of GFP fluorescence, consistent with residual fluorescent GFP expression in differentiating cells (Fig. 6*J*, arrows). Antibody labeling of 7-dpf larvae revealed that the s100β protein was not expressed in radial cells of the PR and LR at this stage, despite strong expression in cellular processes throughout the brain and pituitary (Fig. 6*K*). Thus to examine the radial glial population in relation to Hh-responsive cells at larval stages we crossed the *Tg(gfap:NLS-mCh)* transgenic line to the *Tg((BAC)ptch2:Kaede)* and *Tg(tcfsiam:GFP)* transgenic lines (Fig. 6*L*,*M*). Examination of double transgenic individuals revealed that ∼1/3 of GFAP-expressing radial glia were Hh-responsive at this stage, consistent with the heterogeneity in this population shown above. Conversely, ∼1/3 of Hh-R cells expressed GFAP (Fig. 6*L*). In contrast to Hh-R cells, the majority of Wnt-responsive cells were GFAP negative at this age (Fig. 6*M*), with <2% of Wnt-R cells being double-labeled (Fig. 6*M*, inset).

Together, these results show that at least five embryonic cell-cell signaling systems (Hh, Wnt, FGF, RA, and Notch) persist into adulthood in the zebrafish hypothalamus. Based on spatial expression patterns and co-labeling analysis it appears that at least Shh and Wnt act on distinct populations in the PR, with Shh-producing cells and Notch-responsive cells representing additional distinct cell types. The presence of a small number of co-labeled cells in transgenic larvae containing both the Hh-reporter and the Wnt-reporter suggests that a subset of cells receives signals from these two signaling systems, either simultaneously or sequentially.

### Hh-responsive cells are highly proliferative relative to other hypothalamic radial glia

**Figure 7.**
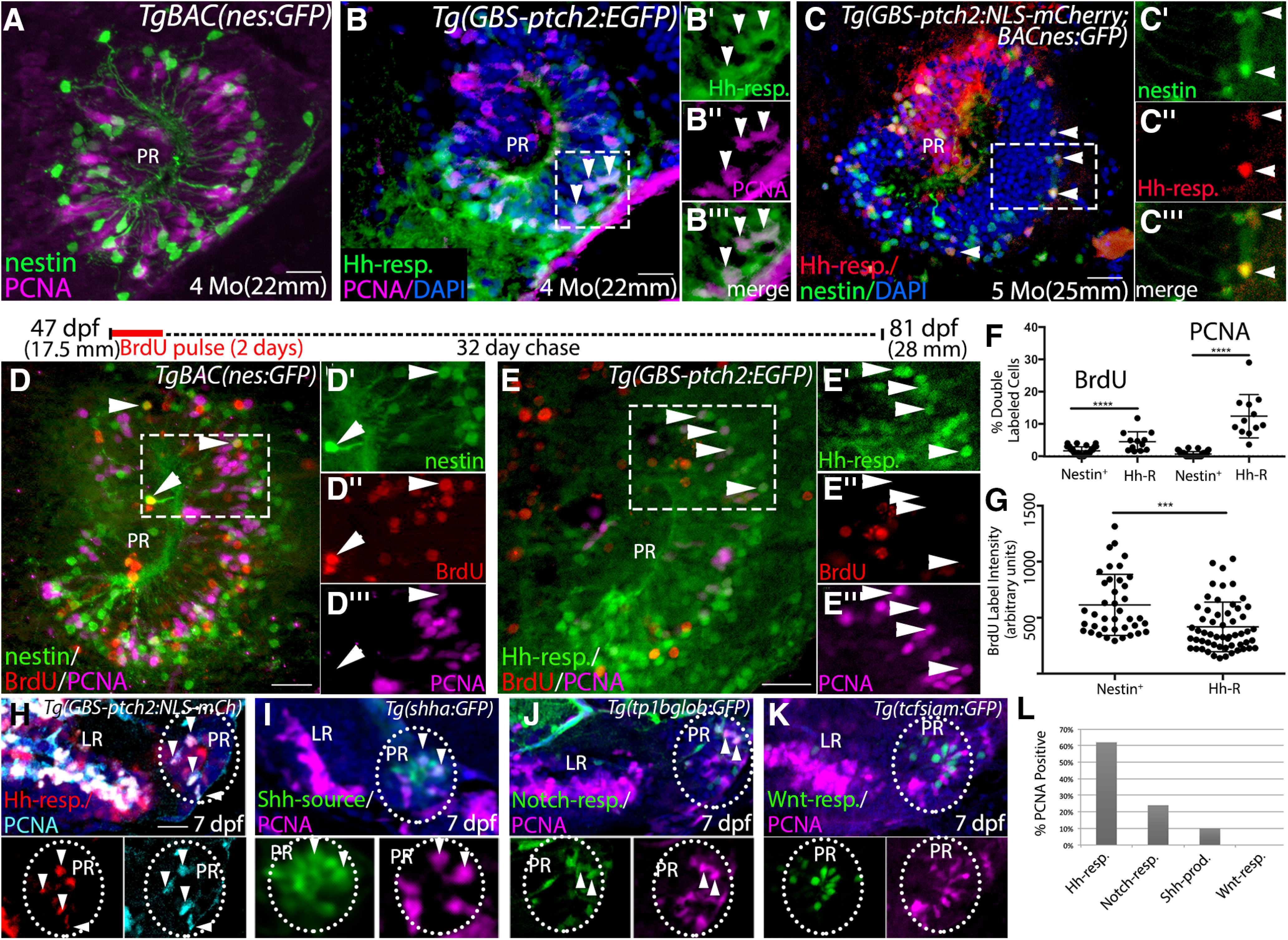
Hh-responsive cells are more highly proliferative than other radial glia in the hypothalamus. ***A***, Sagittal tissue section through the PR of a *TgBAC(nes:EGFP)* transgenic adult brain showing PCNA antibody-labeled proliferative cells. Nestin-expressing cells are predominantly PCNA negative. ***B***, Sagittal tissue section through the PR of a *Tg(ptch2:EGFP)* transgenic adult brain showing PCNA antibody labeled proliferative cells. Arrowheads indicate examples of PCNA-labeled (proliferative) Hh-responsive cells. Panels at right show separated channels from the boxed region. ***C***, Hh-responsive cells (red) are largely distinct from Nestin-expressing cells (green) in the PR of the adult hypothalamus, as visualized in *Tg(GBS-ptch2:NLS-mCherry;nes:EGFP)* double transgenic fish. However, 17% of cells (74 cells of 442 total) contained both GFP and mCherry (arrowheads, *n* = 9 tissue sections from 3 double transgenic fish) with GFP fluorescence substantially lower in double-labeled cells. Panels at right show separated channels from the boxed region. ***D***, ***E***, BrdU pulse-chase experiment. Schematic above panels shows timing of pulse and chase, with 47-dpf adult *TgBAC(nes:EGFP)* or *Tg(GBS-ptch2:EGFP)* fish being exposed to 10 μm BrdU in fish water for 2 d; 32 d later fish were killed, and tissue sections were labeled using anti-BrdU (red) and anti-PCNA (magenta) antibodies. ***D***, Representative sagittal section through the PR of a *TgBAC(nes:EGFP)* adult, insets show single channel data for the boxed region. A small number of Nestin-expressing cells in the PR retained the BrdU label after one month. These cells did not express PCNA (arrowheads), indicating they were not in G_1_/S/G_2_ of the cell cycle at the time of fixation. ***E***, Representative sagittal section through the PR of a *Tg(GBS-ptch2:EGFP)* adult, insets show single channel data for the boxed region. Most Hh-responsive cells failed to retain the BrdU label after one month, and many of these cells expressed PCNA (arrowheads), indicating active cell cycling at the time of fixation. ***F***, Graph showing the percentage of Nestin-expressing or Hh-responsive cells that co-labeled with the BrdU or PCNA antibodies. *Tg(nesGFP)*: *n* = 3 fish, 13–16 tissue sections per fish. *Tg(GBS-ptch2:EGFP)*: *n* = 2 fish, 13–16 sections per fish. ***G***, Quantification of BrdU label intensity in Nestin-expressing and Hh-responsive cells showing BrdU labeling intensity was significantly lower in Hh-responsive cells compared with nestin-expressing cells. ***H–K***, Representative images of PCNA labeling in single confocal optical sections from sagittal sections through the hypothalamus in 7-dpf larvae expressing four different transgenes. Top panels show merged images (transgene reporter + PCNA + DAPI) and bottom panels show single channels. ***H***, In *Tg(GBS-ptch2:NLS-mCh)* larvae over 60% of NLS-mCherry-expressing cells in the hypothalamic ventricular regions are labeled with the PCNA antibody (arrowheads). In the LR, 214 of 288 NLS-mCherry+ cells labeled with PCNA (74%), while in the PR 110 of 117 (62%) cells were double labeled (*n* = 12 sections from 3 larvae). ***I***, Shh producing cells were predominantly found in the PR (dotted oval); 19 of 186 GFP+ cells expressed PCNA (10%, arrowheads; *n* = 15 sections from 3 larvae). ***J***, Notch responsive cells, as revealed in the *Tg(Tp1bglob:GFP)* line, were also localized to the PR (oval); 27 of 113 GFP+ cells were PCNA+ (24%, arrowheads; *n* = 11 sections from 3 larvae). ***K***, Wnt-responsive cells are confined to the PR (oval). None of the 266 cells examined expressed PCNA (*n* = 18 sections from 3 larvae). ***L***, Graph showing the percentage of each transgene-expressing cell type found to also express PCNA; ***p < 0.001, ****p < 0.0001. Source data for graphs can be found in Extended Data Figure 7-1. All panels show 0.5-μm single optical sections of 20-μm tissue sections. Scale bars: 20 μm.

Nestin expression has been used as a hallmark of the transition from quiescent to activated neural stem cell states in the brains of both mammals and teleosts (Wang et al., 2011; Chaker et al., 2016; Obernier and Alvarez-Buylla, 2019). We found that Nestin-expressing radial glia in the adult hypothalamus were largely PCNA negative (Fig. 7*A*,*F*; Extended Data Fig. 7-1), consistent with a quiescent or slow-cycling progenitor state. In contrast, over 10% of Hh-responsive cells in the adult PR were PCNA-positive (Fig. 7*B*,*F*), consistent with an amplifying progenitor state PCNA labeling, which occurs in G1/S/G2 of the cell cycle, was located predominantly toward the basal portion of the adult PR (Fig. 7*A–E*), consistent with G1/S/G2 occurring more distant from the ventricle and M phase occurring apically, as has been shown during neurogenesis in the embryo (Alexandre et al., 2010; Leung et al., 2011). While Hh-responsive cells and Nestin-expressing cells have distinct distributions and proliferative profiles (Fig. 7*A*,*B*), we found that ∼17% of mCherry-expressing (i.e., Hh-responsive) cells in the periphery of the ventricular zone also contained GFP produced from the *nes:GFP* transgene (Fig. 7*C*). Given the perdurance of fluorescent proteins for several days after the cessation of transgene expression (Figs. 3,4; Wang et al., 2012) co-labeling in these cells could reflect either sequential or simultaneous transgene expression in the same cell as it progresses through the stem cell activation-proliferation-differentiation pathway and migrates away from the ventricular zone (Obernier and Alvarez-Buylla, 2019).

10.1523/ENEURO.0226-20.2020.f7-1 Extended Data Figure 7-1 Source data for graphs in Figure 7. Spreadsheet with GFP+, PCNA+, BrdU+ cell quantification and image intensity data used to generate graphs in Figure 7, with exact *p* values. Download Figure 7-1, XLSX file.

To more carefully examine the relative proliferation of nestin-expressing and Hh-responsive radial glia in the adult hypothalamus we performed BrdU pulse-chase experiments on *nes:GFP* and *ptch2:GFP* transgenic fish (Fig. 7*D–F*); 1.5-month-old adults were treated with BrdU for 2 d to label cells passing through S phase, followed by a 32-d chase period. We then used PCNA and BrdU labeling on sagittal through the PR to identify cells that had retained the BrdU label over the one-month chase period and were proliferative at the time of fixation (Fig. 7*D–G*). Consistent with low proliferation rates, very few *nestin*-expressing cells in the PR expressed PCNA (21/3853 cells, <1%). Approximately 2% of these cells retained the BrdU label over the one-month chase period, indicating they had gone through S-phase of the cell cycle one month earlier (Fig. 7*D*,*F*). In contrast, over 10% of Hh-responsive cells in the adult PR were found to be in G1/S/G2 of the cell cycle at the time of fixation based on PCNA expression, while ∼5% of Hh-responsive cells retained the BrdU label, indicating they had progressed through S-phase of the cell cycle one month earlier. Fluorescent intensity of BrdU labeling in these label-retaining Hh-responsive cells was significantly lower than that in the BrdU+/*nestin*+ population (Fig. 7*G*; Extended Data Fig. 7-1), suggesting the Hh-responsive cells had undergone cell division(s) subsequent to the BrdU pulse period, thus reducing the amount of the BrdU label in these cells.

We turned to larval stages to determine the relative proliferation profiles of Hh-responsive, Shh-producing, Notch-responsive, and Wnt-responsive cells in the hypothalamus (Fig. 7*H–L*; Extended Data Fig. 7-1). Approximately 60% of Hh-responsive cells in the larval PR were PCNA-positive, compared with ∼20% of Notch-responsive cells and 10% of Shh-producing cells (Fig. 7*I*,*J*,*L*). We failed to identify any PCNA-positive Wnt-responsive cells in the PR (0/266 cells examined; Fig. 7*K*,*L*), consistent with the low proliferation rate for these cells previously reported (Duncan et al., 2016). These analyses also revealed the LR to be highly proliferative relative to the PR (Fig. 7*H–K*), consistent with the major growth this lobe undergoes relative to the PR during larval stages (e.g., compare Figs. 3*J*, 3 dpf and *D*, 7 dpf and 8*C*, 12 dpf, and 1*J*, adult). Together, these analyses reveal substantial differences in proliferative profiles among progenitors in the PR, with proliferation among the different populations following the order: Hh-responsive > Notch-responsive > Shh-producing > Wnt-responsive. Based on low levels of PCNA and EdU labeling, *nestin*-expressing and Wnt-responsive radial glia represent relatively slow cycling populations while Hh-responsive populations of the hypothalamus represent a much more proliferative population consistent with an amplifying progenitor identity.

### Hh-responsive precursors give rise to dopaminergic, GABAergic, and serotonergic neurons

**Figure 8.**
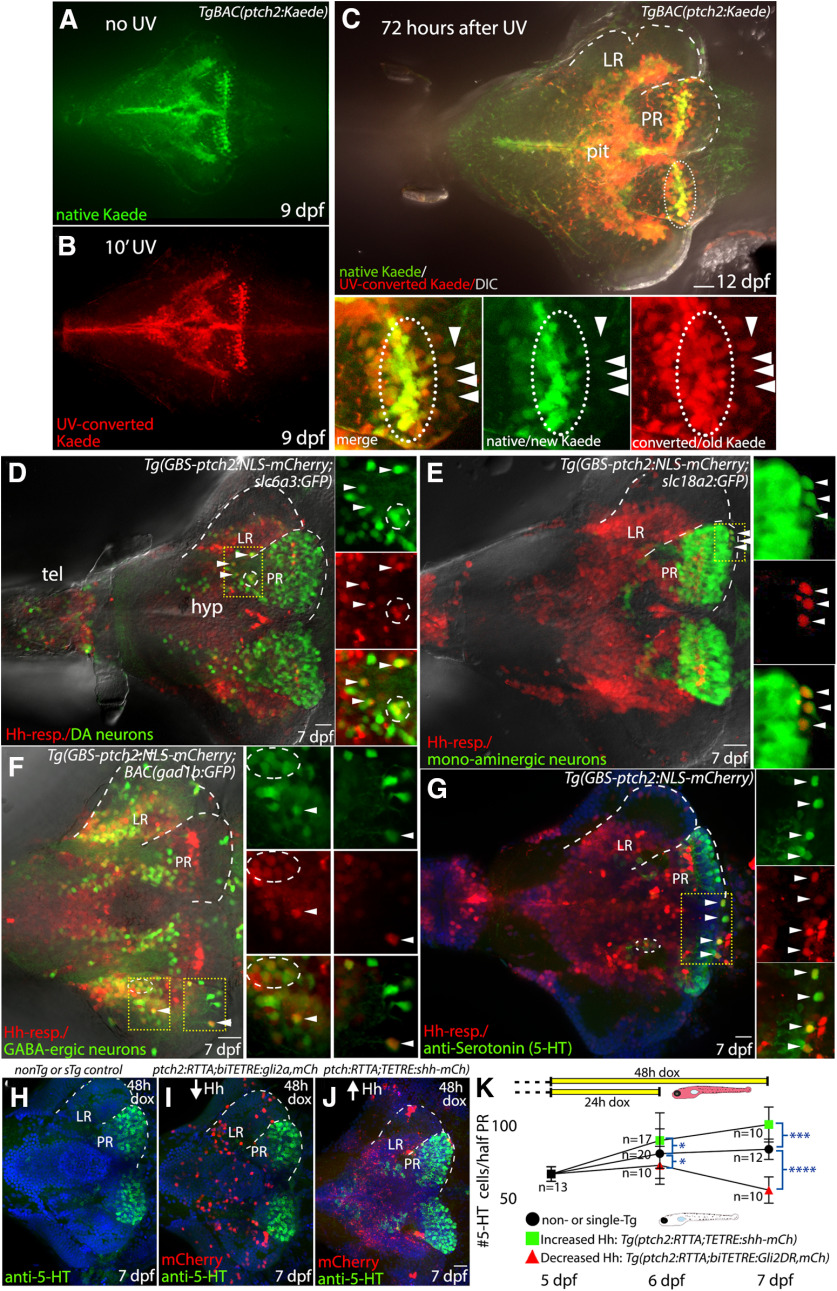
Hh-responsive progenitors of the hypothalamus give rise to dopaminergic, serotonergic, and GABAergic neurons. ***A***, Native (green) Kaede expression in Hh-responsive hypothalamic cells at 9 dpf, as seen in a non-UV-irradiated brain. ***B***, Photoconverted (red) Kaede expression in hypothalamic Hh-responsive cells as seen in the brain from a fish that had been exposed to UV light for 10 min before fixation. ***C***, Kaede expression in the hypothalamus of a fish that had been UV-irradiated 3 d before fixation and imaging. The majority of native/newly produced Kaede protein (green) is seen in the ventricular regions (e.g., inside dotted oval), consistent with continued Hh-target gene expression in proliferative cells. UV-converted Kaede protein (red) is still visible in the ventricular regions 3 d after conversion but is also present in cells more distant from the ventricle that do not contain new/native Kaede protein (arrowheads). Panels at bottom show merged and separated red and green channels as indicated. ***D***, Dopaminergic cells and Hh-responsive cells in the ventral brain, as visualized in a *Tg(slc6a3:EGFP,GBS-ptch2:NLS-mCherry)* double transgenic larva. A small subset of cells expresses both the GFP and the NLS-mCherry proteins (arrowheads, circle), suggesting Hh-responsive cells can give rise to dopaminergic neurons. ***E***, Monoaminergic neurons and Hh-responsive cells as visualized in a *Tg(slc18a2:GFP,GBS-ptch2:NLS-mCherry)* double transgenic larva. Again, a small subset of cells expresses both the GFP and the NLS-mCherry proteins (arrowheads), suggesting Hh-responsive cells can give rise to monoaminergic neurons. ***F***, GABAergic neurons and Hh-responsive cells as visualized in a *TgBAC(gad1b:GFP, GBS-ptch2:NLS-mCherry)* double transgenic larva. A small subset of cells expresses both the GFP and the NLS-mCherry proteins (arrowheads), suggesting Hh-responsive cells can give rise to GABAergic neurons. ***G***, Antibody labeling in a *Tg(GBS-ptch2:NLS-mCherry)* larval brain showing serotonin (5-HT) expression in the ventral hypothalamus. The presence of double-labeled cells is consistent with Hh-responsive cells giving rise to serotonergic neurons. ***H***, ***I***, Ventral views of anti-serotonin antibody labeled 7-dpf larval brains labeled cells following conditional manipulation of Hh signaling using the Tet-On transgenic system. ***H***, Representative single transgenic [*Tg(GBS-ptch2:RTTA-HA)* or *Tg(TETRE:shha-mCherry)*] sibling larva, identified by the lack of mCherry expression, showing number of serotonergic cells in the absence of effector transgene activation. ***I***, Representative *Tg(GBS-ptch2:RTTA,TETRE:shha-mCherry)* double transgenic larva, identified by mCherry expression, showing increased numbers of serotonergic cells in the PR following 2 d activation of the *shha-mCherry* transgene**. *J***, Representative *Tg(GBS-ptch2:RTTA,biTETRE:gli2aDR,NLS-mCherry)* double transgenic larva, identified by mCherry expression, showing decreased numbers of serotonergic cells in the PR following 2 d activation of the *gli2DR* transgene. ***K***, Graph showing serotonergic cell numbers, at 5–7 dpf following 1 or 2 d activation of the Tet-On system in doxycycline (see diagram at top of graph). Error bars indicate SD. Sample numbers for each experimental condition are shown on the graph, with significance determined using a one-way ANOVA; *p < 0.05, ***p < 0.001, ****p < 0.0001. Source data for graphs can be found in Extended Data Figure 8-1. ***A–J***, Ventral views of dissected brains from 7-dpf larvae to show the hypothalamus. Dotted lines outline LR and PR on half of the brain. Small panels at right in ***A–D*** show single channel data for a single optical section in the boxed regions. hyp, hypothalamus; tel, telencephalon. Scale bars: 20 μm.

We next took advantage of a suite of zebrafish transgenic lines to begin to determine the hypothalamic cell types that arise from Hh-responsive precursors. Using the photoconvertible *ptch2:Kaede* line (Huang et al., 2012) we first verified the perdurance of the Kaede protein in the larval hypothalamus (Fig. 8*A–C*). Three days after UV-exposure converted Kaede protein (red) was easily detectable throughout the hypothalamus, revealing the long perdurance of this fluorescent protein. The majority of native/newly produced Kaede protein (green) was present near the ventricle (Fig. 8*C*) consistent with continued active Hh signaling in proliferative cells in the ventricular zones of the LR and PR. We also identified cells outside the ventricular zone that contained only the converted Kaede protein (Fig. 8*C*, arrowheads), indicating progression out of a Hh-responsive state before or just after the UV conversion event 3 d previously. This is consistent with a model in which Hh-responsive progenitors downregulate the Hh response as they move away from the ventricle and differentiate.

10.1523/ENEURO.0226-20.2020.f8-1 Extended Data Figure 8-1 Source data for graphs in Figure 8. Spreadsheet with serotonergic cell quantification data used to generate graph in Figure 8, with exact *p* values. Download Figure 8-1, XLSX file.

To determine whether Hh-responsive progenitors might give rise to dopaminergic, monoaminergic, GABAergic, or glutamatergic neurons, we examined expression of the *ptch2:NLS-mCherry* transgenic fish expressing GFP in these neuronal subtypes. Nuclear mCherry was seen in GFP-expressing dopaminergic neurons as visualized in the *dat:GFP* line (Xi et al., 2011; Fig. 8*D*), in monoaminergic neurons labeled in the *vmat:GFP* line (Wen et al., 2008; Fig. 8*E*) and in GABAergic neurons as visualized in the *gad1b:GFP* line (Satou et al., 2013; Fig. 8*F*). In all cases, co-labeled cells were seen outside the ventricular region and mCherry fluorescence was less intense in co-labeled cells. We did not observe co-labeled cells in *ptch2:NLS-GFP vgluta:DsRed* double transgenic fish, suggesting Hh-responsive cells may not give rise to glutamatergic neurons (data not shown). Finally, anti-serotonin antibody labeling of *ptch2:NLS-mCherry* transgenic larvae identified numerous mCherry positive serotonergic cells, suggesting a current or previous Hh response in this lineage (Fig. 8*F*). Together, these data suggest that Hh-responsive proliferative precursors contribute to dopaminergic, GABAergic, and serotonergic populations in the hypothalamus.

### Hh signaling regulates serotonergic cell numbers in the hypothalamus

To directly examine whether or not Hh/Gli signaling levels influence the hypothalamic serotonergic population, we again employed the Tet-On Hh gene regulation system; 24-h activation of the Gli2DR transcriptional repressor in Hh-responsive cells prevented the normal increase in serotonergic neurons over this time period, with 48-h activation leading to a reduction in serotonergic cell numbers (Fig. 8*K*; Extended Data Fig. 8-1). When compared with age-matched controls (non-transgenic or single-transgenic siblings), Gli2DR-expressing larvae had 10% and 34% fewer serotonergic cells at 6 and 7 dpf, respectively (Fig. 8*H–K*). In contrast, increasing Hh signaling levels through expression of the Shh ligand led to a 11% increase in serotonergic cell numbers after 24 h, and a 21% increase after 48 h of transgene activation (Fig. 8*J*,*K*). Taken together, these data indicate Hh signaling positively regulates the production of at least this neuronal cell type in the hypothalamus.

## Discussion

### Hh signaling regulates neural progenitor proliferation in the hypothalamus

Neurogenesis in the hypothalamus is now known to be required for postembryonic growth, maintenance, and function of this highly conserved vertebrate brain region (Yoo and Blackshaw, 2018). Here, we demonstrate a novel role for the canonical Hh/Smo/Gli signaling pathway in zebrafish hypothalamic precursor proliferation and adult neurogenesis, an apparent extension of its evolutionarily conserved role in establishing the HP axis during embryonic development (Sbrogna et al., 2003; Guner et al., 2008; Devine et al., 2009; Burbridge et al., 2016; Corman et al., 2018). We show that hypothalamic Hh-responsive cells represent a relatively rapidly proliferating multipotent progenitor population in both larval and adult zebrafish, that Hh signaling levels affect proliferation in this population, and that Hh/Gli signaling positively regulates the production of at least serotonergic neurons. Hh/Gli signaling thus appears to be a major regulator of hypothalamic neurogenesis throughout life.

Hh/Gli signaling is well studied as an important regulator of adult neurogenesis in more dorsal regions of the mammalian brain, modulating stem cell renewal and astrocyte differentiation in the subventricular zone of the hippocampus (Machold et al., 2003; Ahn and Joyner, 2005; Ihrie et al., 2011; Álvarez-Buylla and Ihrie, 2014). In the subventricular zone Hh signaling was shown to shorten G_1_ and S-G_2_/M portions of the cell cycle, results that were interpreted as showing a role in neural stem cell activation (Daynac et al., 2016) but are also consistent with a role in progenitor amplification. We show that the Hh-responsive population is highly proliferative relative to other precursors in the hypothalamic niche, suggesting Hh signaling regulates precursor amplification. In larvae, changes in proliferation were seen within 12 h of doxycycline addition using the Tet-On system (Fig. 3). Given the inherent lag time needed for transgene transcription and translation in this system, and combined with the finding that Hh inhibition leads to a loss of *cyclinD* mRNA levels, these data point to a rapid role for Hh signaling in positively regulating cell cycle progression, providing a mechanism for controlling growth rates in larvae and the production of new neurons and glia in adults. The fact that a substantial number of adult Hh-responsive cells retained BrdU label for more than a month also revealed substantial heterogeneity in cell cycle kinetics within the hypothalamic precursor population.

The zebrafish brain undergoes rapid growth during larval stages, with some brain regions growing more rapidly than others as the brain takes on its mature morphology (Fig. 1*A*). Within the larval hypothalamus, the LR appears to be a region of relatively rapid growth as it extends laterally and posteriorly to eventually surround the hypothalamic lobe that contains the PR (compare Figs. 3*J*, 3 dpf, and *D*, 7 dpf, and 8*A*, 12 dpf, and 1*A*, adult). In larvae, the LR contains relatively more proliferative Hh-responsive cells than the PR (Fig. 6*F-H*; data not shown), consistent with Hh signaling helping regulate progenitor amplification as part of differential regional growth. While neurogenesis continues throughout life in the zebrafish brain (Chapouton et al., 2007; Kizil et al., 2012; Schmidt et al., 2013), brain growth slows dramatically after larval stages. In three-month-old adults, the percentage of Hh-responsive cells that express PCNA dropped from 60% to ∼10%, coincident with the slower growth rates seen in early adulthood. The Hh/Gli signaling system remains active in a smaller number of hypothalamic cells in the PR in nine-month-old fish, with this response restricted to the midline and PR (Fig. 1), suggesting an ongoing role in tissue maintenance and possibly function.

### The hypothalamic PR: a complex neural progenitor niche regulated by multiple signaling molecules

The complex combinations of extrinsic factors that regulate the activation, amplification, and differentiation of neural precursors within stem cell niches in different regions of the brain remain poorly understood (Urbach and Witte, 2019). In mammals, Hh, Notch, and Wnt signaling systems have all been shown to remain active in the adult hypothalamus (Mirzadeh et al., 2017) and FGF signaling is linked to postnatal hypothalamic proliferation, energy balance, and appetite (Robins et al., 2013). We and others have now shown that Wnt, Hh, Notch, FGF, and RA signaling systems are all active in the hypothalamic ventricular zone of larval and adult zebrafish (this study; Guner et al., 2008; Topp et al., 2008; Wang et al., 2009; Shearer et al., 2010), indicating this region may represent a complex and heterogeneous signaling environment similar to mammalian stem cell niches (Chaker et al., 2016).

The availability of zebrafish reporter lines that express fluorescent proteins in different progenitor populations allowed us to examine the cellular morphology and spatial relationships of cells responding to Hh, Wnt, and Notch signaling. Nestin-expressing radial glia have been identified as neurogenic cells in the zebrafish telencephalon (Chapouton et al., 2007; Kaslin et al., 2009; Ganz et al., 2010) and Nestin expression is thought to be a hallmark of the transition from quiescent to activated neural stem cells in both mammals and teleosts (Wang et al., 2011; Chaker et al., 2016; Obernier and Alvarez-Buylla, 2019). Consistent with a quiescent/activated stem cell identity in the hypothalamic niche, we found that only a small percentage of hypothalamic Nestin-expressing cells expressed PCNA. A small percentage of Hh-responsive precursors contained both mCherry and GFP in *ptch2:NLS-mCherry*,*nes:GFP* double transgenic animals. Given the perdurance of fluorescent proteins for several days in cells, this is consistent with the Hh-transcriptional response beginning in cells that had previously downregulated Nestin expression. Alternatively, this co-labeling could indicate that Hh signaling Hh may play a role in activating this less-proliferative population.

Our studies revealed dramatically different proliferative profiles among hypothalamic progenitor populations that are transcriptionally responsive to Hh, Notch, and Wnt signaling. Based on PCNA labeling, Hh-responsive cells were the most highly proliferative at all stages examined. Wnt signaling was shown previously to play an important role in hypothalamic neurogenesis within the PR in zebrafish (Wang et al., 2009, 2012), likely regulating differentiation events rather than proliferation (Duncan et al., 2016). Consistently, our PCNA analysis revealed Wnt-responsive cells to be largely non-proliferative (Fig. 7*K*). In other systems, Hh and Wnt signaling pathways are known to drive proliferation in the same cells (Alvarez-Medina et al., 2009). The fact that Wnt-responsive cells in the PR are largely non-proliferative, combined with our finding that only a small percentage of cells co-label in the Wnt-reporter and Hh-reporter transgenic lines, is consistent with a model in which Wnt signaling acts to drive differentiation of a subset of Hh-responsive amplifying cells in the hypothalamic niche. Alternatively, Wnt and Hh signaling may act on distinct precursors, or act sequentially at earlier stages of the stem cell activation-amplification-differentiation pathway, with Wnt acting on the early activation of quiescent cells followed by Hh regulating the level of progenitor amplification before differentiation. Additional studies will be needed to determine whether or not these different signaling systems sequentially cycle through a given cell cycle and how possible oscillations in signal responses change as the brain matures and ages.

In the zebrafish and mammalian telencephalon, Notch signaling is thought to act to keep stem cells in a quiescent state, with inhibition of Notch signaling leading to the activation of these cells as a first step in the stem cell proliferation pathway (Carlén et al., 2009). We show that Notch signaling is active in the hypothalamic niche, with the Notch-responsive population having a proliferative profile intermediate between Hh-responsive and Wnt-responsive progenitors. While further investigation is needed, based on relative cell numbers, relative proliferation rates, and the distribution of these cell types, it is possible that Notch signaling may regulate early steps (e.g., activation) in hypothalamic stem cell progression as seen in the telencephalon (Chapouton et al., 2010), with Hh/Gli then acting further downstream to control proliferation rates and Wnt-signaling driving differentiation (Palma et al., 2005; Locker et al., 2006; Alvarez-Medina et al., 2009; Duncan et al., 2016; Corman et al., 2018). The results presented here set the stage for detailed analyses of how these multiple signaling pathways combine to regulate stem cell activation, proliferation, and differentiation of distinct neural stem cell populations within the vertebrate hypothalamus.

### Adult neurogenesis and hypothalamic function

A number of studies have now linked proliferation in the hypothalamus to distinct hypothalamic functions, including regulation of energy metabolism in rodents (Kokoeva et al., 2005, 2007; Lee et al., 2012), anxiety in zebrafish (Xie et al., 2017), and seasonal reproductive changes in sheep (Migaud et al., 2011). Each of these functions requires accurate integration of body state with external cues from the environment, with the precise coordination of proliferation and neurogenesis potentially involved in both acute and long-term adaptation (Yoo and Blackshaw, 2018). Since HP axis homeostatic functions rely on the regulated output of a large number neurosecretory cells, regulating cell numbers of distinct populations may be an important component of normal homeostasis. Shh and other embryonic cell-cell signaling systems that are active in the hypothalamic niche are thus positioned as possible mediators of short-term plasticity in neuronal populations that contributes to homeostasis. In this study we show a role for Hh signaling in regulating at least hypothalamic serotonergic cell numbers. The data presented here add to a growing body of evidence that link embryonic cell-signaling systems to the regulation of mature neuronal populations that is needed for life-long homeostasis and metabolic health, with coordinated mitogenic and differentiation signals among multiple signaling systems potentially underlying adult hypothalamic plasticity critical for the vertebrate response to metabolic challenges.

## Synthesis

Reviewing Editor: Katalin Toth, Universite Laval Decisions are customarily a result of the Reviewing Editor and the peer reviewers coming together and discussing their recommendations until a consensus is reached. When revisions are invited, a fact-based synthesis statement explaining their decision and outlining what is needed to prepare a revision will be listed below.

This is a thorough study examining progenitor cells in the zebrafish and the role of Hh in the regulation of neurogenesis. The conclusions are based on technically solid data sets. Both reviewers were very positive about the importance of the findings and the strengths of the authors’ conclusions. However, they both identified several points where the manuscript could be improved, these points should be carefully addressed by the authors.

Rev. #1.

In this manuscript the authors examine neurogenesis in the adult zebrafish hypothalamus. The goal was to examine various cell populations that undergo neurogenesis and the cellular factors that might underlie the heterogeneity of these populations. The authors examined proliferating radial glia cells that are responsive to hedgehog, Notch and Wnt and that express Sonic Hedgehog. The manuscript is very well written with few grammatical errors. The authors do an excellent job of examining cell populations with appropriate controls and confirmatory experiments. The experiments are well-constructed and provide firm results that highlight the existence of multiple populations of cells that give rise to dopaminergic, serotonergic and GABA-ergic, but no glutamatergic neurons. I only have a few minor comments as follows:

1) Results, line 232-236: the authors performed intraperitoneal injection of Cya into adults followed by BrdU assay 24 hours later. Proliferation was reduced at this point. Was proliferation

2) Results, line 199: should read “...ventricle includes two recesses...”

3) Results, line 200-201: the term “lateral recess” is lower case while in the next line “Posterior Recess” is capitalized. Please be consistent with capitalizations.

4) Results, line 206: should probably read “...accurately reflected in the ptch2 and ...”

5) Results, line 329: should read “...has been used as a hallmark...”

6) Discussion line 430: should read “Neurogenesis in the hypothalamus...”

Rev. #2.

This manuscript represents a careful and thorough analysis of the various progenitor cell populations present in the larval and adult zebrafish hypothalamus. The evidence that Hh regulates postembryonic hypothalamic neurogenesis in this species confirms the notion of a conserved role in vertebrates and the extent of progenitor cell heterogeneity with respect to responsiveness to signalling pathways is novel. Very good use of transgenic and endogenous marker analyses to examine the extent of Hh signalling and its modification with loss and gain of function.

Major comments:

(1) In general, it would be useful to have baseline data on how many Hh-resp cells are present in the LR and PR and how may are radial glial cells (e.g., co-label with S100b+, GFAP). This could be said about the other signal-responsive cells as well. A major conclusion of this study is that the LR and PR neurogenic niches are heterogeneous, but it’s not clear if this is heterogeneity of RG cells specifically and/or other types of progenitors.

(2) Hh-resp cells in the PR are described as “multipotent” but this has not been determined at a clonal level. Therefore, as a population these cells produce various neuronal subtypes of cells, but whether a single Hh-responsive cell gives rise to all neuronal subtypes cannot be assumed in this type of analysis. The authors should qualify these interpretations more carefully in the discussion.

(3) I’m a little confused about the findings in Fig 3L and M. Why are there no “yellow” cells in the LR and PR? Increased signalling should promote increased proliferation in the cells that have the increased signalling. If these Hh-resp cells are not the ones proliferating, then Hh signalling is indirectly affecting the proliferation of other cells

(4) Related to point 3 above (lack of apparent double positive cells), are the number of proliferating cells (EdU+) from the experiments presented in Fig 3I-N representative of the mCherry+ fraction or just total EdU+ cells per structure? This is very important to clarify because it would influence the interpretation of direct versus indirect Hh signalling affecting cell proliferation.

Minor points:

(1) Abstract - reference to the “rapidity” of proliferation seems out of place since there were no cell cycle analyses comparing the different populations, only an estimate the fraction of proliferating cells in each population

(2) In Fig 6, to what extent does the multiple signalling pathways oscillate with cell cycle and age? The conclusion from these analyses is that there is extensive stable heterogeneity, but these caveats should at least be acknowledged in the Discussion.

(3) In Fig 6F-H the degree of heterogeneity with this analysis is somewhat overstated because combinatorial co-labeling of the other pathway responsive cells was not exhaustive. Perhaps this can be qualified.

(4) Not clear why the differences in proliferative profiles for radial cells responsive to different signalling pathways is “striking” (lines 375-376).

## Author Response

Authors Response to Reviewers’ Comments

We appreciate the reviewers’ positive evaluation of our work and the thorough comments and suggestions that help improve the manuscript. In response we have now revised the manuscript substantially (changes indicated by blue text) and address all reviewer concerns point-by-point below. To address reviewer #2’s first major concern we have added substantial new data showing the proportions of the radial glial population in the hypothalamic ventricle that are Hh-responsive and Wnt-responsive (new panels J-M of Figure 6). We have also added new images showing that a subset of cells induced to express the Gli1 transcription factor (using the Tet-On system) are EdU positive (new insets in Figure 3M). These data strongly support the proposed model for cell autonomous action of Hh signaling in regulating progenitor proliferation.

Rev. #1.

In this manuscript the authors examine neurogenesis in the adult zebrafish hypothalamus. The goal was to examine various cell populations that undergo neurogenesis and the cellular factors that might underlie the heterogeneity of these populations. The authors examined proliferating radial glia cells that are responsive to hedgehog, Notch and Wnt and that express Sonic Hedgehog. The manuscript is very well written with few grammatical errors. The authors do an excellent job of examining cell populations with appropriate controls and confirmatory experiments. The experiments are well-constructed and provide firm results that highlight the existence of multiple populations of cells that give rise to dopaminergic, serotonergic and GABA-ergic, but no glutamatergic neurons. I only have a few minor comments as follows:

1) Results, line 232-236: the authors performed intraperitoneal injection of Cya into adults followed by BrdU assay 24 hours later. Proliferation was reduced at this point. Was proliferation reduced at even earlier time points, and if so, how soon did it occur?"

We absolutely agree that the kinetics of Hh action is an important issue. Unfortunately, we do not have data for the shorter drug exposures in adults requested by the reviewer. This question is particularly challenging to address in adults for practical reasons (including uncertainties in the kinetics of drug action as well as the minimum time needed for BrdU delivery), and because this in vivo proliferative population is heterogeneous/non-synchronous, as would be expected. To solve some of the practical issues we turned to the larval studies described in the manuscript that point to a rapid effect on the cell cycle (<12 hours). Further studies are underway to better pinpoint the exact kinetics of Hh action, but these necessitate tools and more refined techniques under development in the lab.

To address this reviewer comment we have expanded on our discussion of findings that point to a rapid effect of Hh on progenitor proliferation. We have added a sentence on the timing of transgene activation in the results section (line 270), highlighting the rapid effect on the already rapid larval cell cycle shown in figure 3I-N. We have also expanded the discussion of the kinetics of Hh action on the cell cycle (line 474).

2) Results, line 199: should read “...ventricle includes two recesses...”

This has been corrected (now line 198).

3) Results, line 200-201: the term “lateral recess” is lower case while in the next line “Posterior Recess” is capitalized. Please be consistent with capitalizations.

This has been corrected throughout the manuscript.

4) Results, line 206: should probably read “...accurately reflected in the ptch2 and ...”

This has been corrected (now line 205).

5) Results, line 329: should read “...has been used as a hallmark...”

This has been corrected (now line 352).

6) Discussion line 430: should read “Neurogenesis in the hypothalamus...”

This has been corrected, now line (454)

Rev. #2.

This manuscript represents a careful and thorough analysis of the various progenitor cell populations present in the larval and adult zebrafish hypothalamus. The evidence that Hh regulates postembryonic hypothalamic neurogenesis in this species confirms the notion of a conserved role in vertebrates and the extent of progenitor cell heterogeneity with respect to responsiveness to signalling pathways is novel. Very good use of transgenic and endogenous marker analyses to examine the extent of Hh signalling and its modification with loss and gain of function.

Major comments:

(1) In general, it would be useful to have baseline data on how many Hh-resp cells are present in the LR and PR and how may are radial glial cells (e.g., co-label with S100b+, GFAP). This could be said about the other signal-responsive cells as well. A major conclusion of this study is that the LR and PR neurogenic niches are heterogeneous, but it’s not clear if this is heterogeneity of RG cells specifically and/or other types of progenitors.

Thanks to this suggestion we have now established a baseline for the percentage of Hh-responsive and Wnt-responsive cells that are radial glia (and vice versa) using the radial glial markers S100ß and GFAP (Figure 6, new panels J-M). A new paragraph starting on line 324 now describes the additional analysis, supporting and adding to our characterization of precursor heterogeneity in the hypothalamus.

(2) Hh-resp cells in the PR are described as “multipotent” but this has not been determined at a clonal level. Therefore, as a population these cells produce various neuronal subtypes of cells, but whether a single Hh-responsive cell gives rise to all neuronal subtypes cannot be assumed in this type of analysis. The authors should qualify these interpretations more carefully in the discussion.

This is an important point and we have removed the term multipotent in favor of more direct descriptions of the cell types that arise from this population. We retain the use of multipotent on line 439 (now 461) as it refers to the population rather than individual cells.

(3) I’m a little confused about the findings in Fig 3L and M. Why are there no “yellow” cells in the LR and PR? Increased signalling should promote increased proliferation in the cells that have the increased signalling. If these Hh-resp cells are not the ones proliferating, then Hh signalling is indirectly affecting the proliferation of other cells.

We appreciate the reviewer pointing out that this outcome was not adequately addressed in the manuscript. In figure 3M we now point to EdU labeling in a subset of cells expressing the Gli1-mCherry transgene, consistent with cell autonomous activation of proliferation in these cells. Cells that ectopically express the secreted Shh ligand would not necessarily be expected to be proliferative themselves, and we did not identify double-labeled cells in this case (Fig. 3L). Similarly, cells expressing the Gli2 dominant repressor protein did not incorporate EdU (Fig. 3K), consistent with this transcriptional repressor autonomously preventing cell-cycle progression. Text describing this finding has been added to the results (line 276) and the Figure 3 legend (line 962).

(4) Related to point 3 above (lack of apparent double positive cells), are the number of proliferating cells (EdU+) from the experiments presented in Fig 3I-N representative of the mCherry+ fraction or just total EdU+ cells per structure? This is very important to clarify because it would influence the interpretation of direct versus indirect Hh signalling affecting cell proliferation.

Quantification of EdU labeling was performed for the entire hypothalamus (LR and PR) and was not restricted to transgene-expressing cells. This is now indicated in the graph label and the legend for Figure 3 (line 965).

Minor points:

1) Abstract - reference to the “rapidity” of proliferation seems out of place since there were no cell cycle analyses comparing the different populations, only an estimate the fraction of proliferating cells in each population

We agree that this is an important distinction and we have now replaced “rapidly proliferating...” with “highly proliferative precursor population” (line 12).

(2) In Fig 6, to what extent does the multiple signalling pathways oscillate with cell cycle and age? The conclusion from these analyses is that there is extensive stable heterogeneity, but these caveats should at least be acknowledged in the Discussion.

This is a key question. Unfortunately, we do not have an answer at this time due to a lack of real-time/temporal data. While we began to touch on the question of signal interactions during stem cell cycle in the discussion (e.g. paragraph beginning line 503), we have now expanded on this to add a caveat to the discussion as suggested by the reviewer (line 544).

(3) In Fig 6F-H the degree of heterogeneity with this analysis is somewhat overstated because combinatorial co-labeling of the other pathway responsive cells was not exhaustive. Perhaps this can be qualified.

While distinct expression patterns strongly suggest that each of these signaling systems acts on distinct (although likely overlapping) populations, we agree that the idea of heterogeneity needs to be qualified, especially in light of comment #2 above. We have thus qualified this conclusion in several ways:

1) In the legend describing the signaling schematic (Fig. 6) we now state, “Note that the data do not rule out the possibility that subsets of different signal-responsive cells may overlap both spatially and temporally.”

2) We have rewritten the paragraph in the results section starting on line 343 as follows:

"Together, these results show that at least five embryonic cell-cell signaling systems (Hh, Wnt, FGF, RA, and Notch) persist into adulthood in the zebrafish hypothalamus. Based on spatial expression patterns and co-labeling analysis it appears that at least Shh and Wnt act on distinct populations in the PR, with Shh-producing cells and Notch-responsive cells representing additional distinct cell types. The presence of a small number of co-labeled cells in transgenic larvae containing both the Hh-reporter and the Wnt-reporter suggests that a subset of cells receives signals from these two signaling systems, either simultaneously or sequentially. “

(4) Not clear why the differences in proliferative profiles for radial cells responsive to different signalling pathways is “striking” (lines 375-376).

We agree this is subjective and have replaced “striking” with “substantial” (now line 400).
